# Using Dual Regression to Investigate Network Shape and Amplitude in Functional Connectivity Analyses

**DOI:** 10.3389/fnins.2017.00115

**Published:** 2017-03-13

**Authors:** Lisa D. Nickerson, Stephen M. Smith, Döst Öngür, Christian F. Beckmann

**Affiliations:** ^1^Applied Neuroimaging Statistics Lab, McLean HospitalBelmont, MA, USA; ^2^Department of Psychiatry, Harvard Medical School, Harvard UniversityBoston, MA, USA; ^3^Nuffield Department of Clinical Neurosciences, Oxford University Centre for Functional MRI of the Brain, John Radcliffe Hospital, University of OxfordOxford, UK; ^4^Schizophrenia and Bipolar Disorder Research Program, McLean HospitalBelmont, MA, USA; ^5^Department of Cognitive Neuroscience, Radboud University Medical CentreNijmegen, Netherlands; ^6^Center for Cognitive Neuroimaging, Donders Institute for Brain, Cognition and Behavior, Radboud UniversityNijmegen, Netherlands

**Keywords:** ICA, dual regression, resting state networks, functional connectivity, amplitude

## Abstract

Independent Component Analysis (ICA) is one of the most popular techniques for the analysis of resting state FMRI data because it has several advantageous properties when compared with other techniques. Most notably, in contrast to a conventional seed-based correlation analysis, it is model-free and multivariate, thus switching the focus from evaluating the functional connectivity of single brain regions identified a priori to evaluating brain connectivity in terms of all brain resting state networks (RSNs) that simultaneously engage in oscillatory activity. Furthermore, typical seed-based analysis characterizes RSNs in terms of spatially distributed patterns of correlation (typically by means of simple Pearson's coefficients) and thereby confounds together amplitude information of oscillatory activity and noise. ICA and other regression techniques, on the other hand, retain magnitude information and therefore can be sensitive to both changes in the spatially distributed nature of correlations (differences in the spatial pattern or “shape”) as well as the amplitude of the network activity. Furthermore, motion can mimic amplitude effects so it is crucial to use a technique that retains such information to ensure that connectivity differences are accurately localized. In this work, we investigate the dual regression approach that is frequently applied with group ICA to assess group differences in resting state functional connectivity of brain networks. We show how ignoring amplitude effects and how excessive motion corrupts connectivity maps and results in spurious connectivity differences. We also show how to implement the dual regression to retain amplitude information and how to use dual regression outputs to identify potential motion effects. Two key findings are that using a technique that retains magnitude information, e.g., dual regression, and using strict motion criteria are crucial for controlling both network amplitude and motion-related amplitude effects, respectively, in resting state connectivity analyses. We illustrate these concepts using realistic simulated resting state FMRI data and *in vivo* data acquired in healthy subjects and patients with bipolar disorder and schizophrenia.

## Introduction

Spatial independent component analysis (ICA) has emerged as a popular tool for investigating brain network functional connectivity measured using functional magnetic resonance imaging (FMRI) (McKeown et al., 1998; Cordes et al., 1999; Calhoun et al., 2001; Kiviniemi et al., 2003; Greicius et al., 2004; van de Ven et al., 2004; Beckmann et al., 2005). This technique aims to identify underlying hidden spatio-temporal processes that co-occur to give rise to the measured blood oxygenation level dependent (BOLD) FMRI signals from the brain. These spatio-temporal processes can correspond to spontaneous fluctuations in resting state networks (RSNs) during wakeful rest, to task-locked alterations in brain networks during task performance, to scanner artifacts that occur during the imaging period, or to physiological noise. The appeal of the spatial ICA is that no time-series model of brain activity is required for the analysis—in that sense, it is model-free—and as a consequence, the spatial ICA has the potential to identify spatio-temporal signal sources that are not well-characterized or well-understood. These properties are well-suited to the analysis of FMRI measurements using a resting state paradigm in which there is no task *per se* (for example, resting with eyes open or closed, during sleep, or during pharmacologic manipulations) to investigate the resting state functional connectivity (RSFC) of brain networks.

The standard approach for investigating RSFC of brain networks implements a multi-subject analysis in which a group-average spatial ICA, or group ICA (GICA), is done on the entire set of FMRI data concatenated across all subjects (Calhoun et al., 2001; Beckmann and Smith, 2004). This technique will identify the collection of networks that are common to the entire set of subjects. Because the output spatial maps are common to all subjects, further processing is necessary to compare functional connectivity of any given network between groups of subjects or conditions. For this purpose, the subject-specific network maps corresponding to each group ICA map must be identified in each subject, similar to a contrast map from a task FMRI analysis. These subject-specific network maps capture between-subject variability in the “shape,” or spatial pattern, of the network and can be used in a higher-level general linear model analysis to investigate group differences in functional connectivity.

In addition to the shape of an RSN, the amplitude of an RSN (e.g., the magnitude of the BOLD activity in the RSN) has been shown to convey important information regarding resting state activity. For example, positron emission tomography (PET) task activation studies have long reported decreases in cerebral blood flow (CBF) in a certain collection of brain regions when comparing task to passive rest conditions (Raichle et al., 2001). This collection of brain regions also has high cerebral metabolic rate of oxygen consumption (CMRO_2_) and CBF at rest and, and even early on, the magnitude of the decreases in CBF during brain activation were noted to be likely related to task difficulty, suggesting that magnitude of deactivation carries important information (Shulman et al., 1997). This collection of brain regions is now well-known as the default mode network (DMN), the physiology of which has been studied extensively using PET (Gusnard and Raichle, 2001; Raichle and Snyder, 2007). The function of the DMN has also been studied extensively with BOLD FMRI during wakeful rest (Greicius et al., 2003) using the seed-based connectivity analysis approach first presented by Biswal et al. (1995), and using ICA-based approaches (Beckmann et al., 2005; Smith et al., 2009) and other methods (Andrews-Hanna et al., 2010). BOLD FMRI depends on CBF, CMRO_2_, and cerebral blood volume, thus results from PET studies would also predict effects on BOLD signal amplitudes in the DMN during task performance.

Importantly, now several studies have shown that the amplitude of BOLD activity within the DMN, measured as the standard deviation of the BOLD signal timecourse, is related to task-load during brain activation (McKiernan et al., 2003; Singh and Fawcett, 2008) and is sensitive to different resting state conditions (eyes open with and without fixation vs. eyes closed; Yan et al., 2009 using the power spectrum). Other brain regions also show similar amplitude-related effects. For example, Bianciardi et al. (2009) and Jao et al. (2013) have shown that the amplitude of resting state BOLD signal oscillations in the visual cortex is smaller with an eyes-open fixation resting condition relative to resting with eyes closed. Jao et al. (2013) also reported differences in amplitude of BOLD signals from nodes of the DMN during these two states. Resting FMRI amplitudes can be affected by pharmacologic stimulation (Kiviniemi et al., 2005), by disease (Zang et al., 2007; Vargas et al., 2013) and can also reliably predict task FMRI responses in activated brain regions, for example, during motor and breath hold tasks (Kannurpatti et al., 2012). In addition, Kannurpatti and Biswal (2008) proposed the use of resting state fluctuation amplitudes to scale task-induced BOLD responses for calibrated BOLD, as a proxy for breath holding or 5% CO_2_ inhalation. Thus, the amplitude of the resting FMRI signal likely also reflects an important aspect of RSFC and preserving such amplitude information within any inferential procedure is important to more fully characterize functional connectivity within and between subjects.

The seed-based correlation approach (SBCA) for investigating RSFC originally relied on simple time series correlation (to detect similarities, e.g., synchronous fluctuations, between the timecourse from a seed-region and the timecourses from different parts of the brain). However, it is now common practice to implement SBCA as a voxel-wise multiple regression analysis that is capable of handling confounders such as physiological and subject motion. SBCA will reveal the full connectivity profile of a given seed region, showing all of the brain regions that are functionally connected to the seed in the connectivity map. As a result, it is challenging to differentiate the distinct networks with which a seed region may be functionally connected. One would have to visually “parse” in a purely qualitative way those blobs out into separate networks for interpretation in the context of which networks were connected with the seed. In addition, network amplitude effects will not be accurately reflected using such an approach, with (voxel-wise estimated) correlation coefficients not reflecting amplitude at all (all other things being equal), and regression coefficients in this framework being estimated relative to the seed-timecourse amplitude itself. To disentangle functional connectivity and amplitude of multiple networks, in e.g., the repertoire of brain networks known to be “active” at rest in the human brain (Beckmann et al., 2005; Smith et al., 2009), multivariate regression approaches that analyse the behavior of all networks simultaneously, for example, dual regression [DR; Beckmann et al., 2009; Filippini et al., 2009; implemented in FMRIB Software Library (FSL)], are most suitable. In this case, a full set of brain network maps derived from a GICA are used with DR to derive the subject-specific spatial maps and timecourses corresponding to each GICA spatial map, which maintains sensible control over the contribution of amplitude effects while also providing information regarding network shape and disentangling the effects of multiple networks. Crucially, motion effects can also be a source of amplitude effects by causing abrupt and potentially large spikes or discontinuities in FMRI timeseries, introducing variance into the timecourse that can mimic an amplitude effect. Many studies have shown that motion is a major source of variability in functional connectivity studies that can lead to erroneous results when comparing groups of subjects (Power et al., 2012; Satterthwaite et al., 2012; Van Dijk et al., 2012). Thus, it is crucial to investigate functional connectivity within a framework that maintains control over any amplitude effects that may be present in the data, whether real network effects or motion-related.

In this report, we investigate the use of FSL's group ICA with dual regression (GICA-DR) approach for assessing group/condition differences in functional connectivity. In contrast to group SBCA, there is no need for specifying either subject-specific seed timecourses from regions of interest or confound regressors to capture subject-specific noise effects in the FMRI data. Instead, the GICA identifies the spatiotemporal signals associated with RSNs (e.g., networks of interest), artifacts, and noise that are inherent in the FMRI data. It does so by decomposing the FMRI data in a purely data-driven way into a set of independent components (ICs), with each IC consisting of a spatial map (that is statistically independent from the other IC maps), and a corresponding timecourse. For resting state data, the IC timecourses from a GICA are generally not interpretable because it is common to do data reduction prior to the GICA. However, the spatial maps identified from GICA characterize RSNs that are common to all the subjects, providing a more comprehensive view of the functional hierarchy of the brain (Beckmann et al., 2005), while also identifying artifacts that are, in principle, being identified as components separate from RSNs. Although there are some methodological challenges to ICA (Cole et al., 2010) there is now a consensus that spatial ICA is a powerful analytical tool for investigating functional connectivity across multiple functional domains, such as sensory, motor, cognitive, and limbic systems, simultaneously. Once the group IC maps have been identified by GICA, DR is used to estimate the subject-specific network timecourses and the subject-specific spatial maps for each corresponding GICA map. The resulting subject-specific timecourses and spatial maps can be used to compare functional connectivity between groups/conditions. Some studies have discussed or compared SBCA and ICA-based approaches for assessing group differences in brain network functional connectivity (Cole et al., 2010; Van Dijk et al., 2010; Smith et al., 2014) and have investigated the test-retest reliability and other aspects of GICA-DR (Zuo et al., 2010; Allen et al., 2012). In this study, we focus on how to use GICA-DR to investigate RSN shape while maintaining control over amplitude effects.

### Group ICA with dual regression (GICA-DR)

The GICA identifies a set of IC maps that are common to the entire population (e.g., FMRI data from every subject is included in the analysis) and the dual regression is a mathematical tool that utilizes these IC maps as network templates to identify the corresponding functional connectivity maps in each subject. Although it is generally recommended to use the output spatial maps of a GICA of the dataset under investigation in the DR, dual regression is a generic method in the sense that it can be used to assess the functional connectivity of networks (or parcellations) that are identified using any strategy—not just a GICA of the FMRI data. For example, a set of template networks could be derived from a GICA of an independent set of subjects, from an atlas, or using functional localizers, although these templates may not contain information related to specific artifacts or sources of noise in the data under study, as would a GICA of the original data.

**Figure 1:**
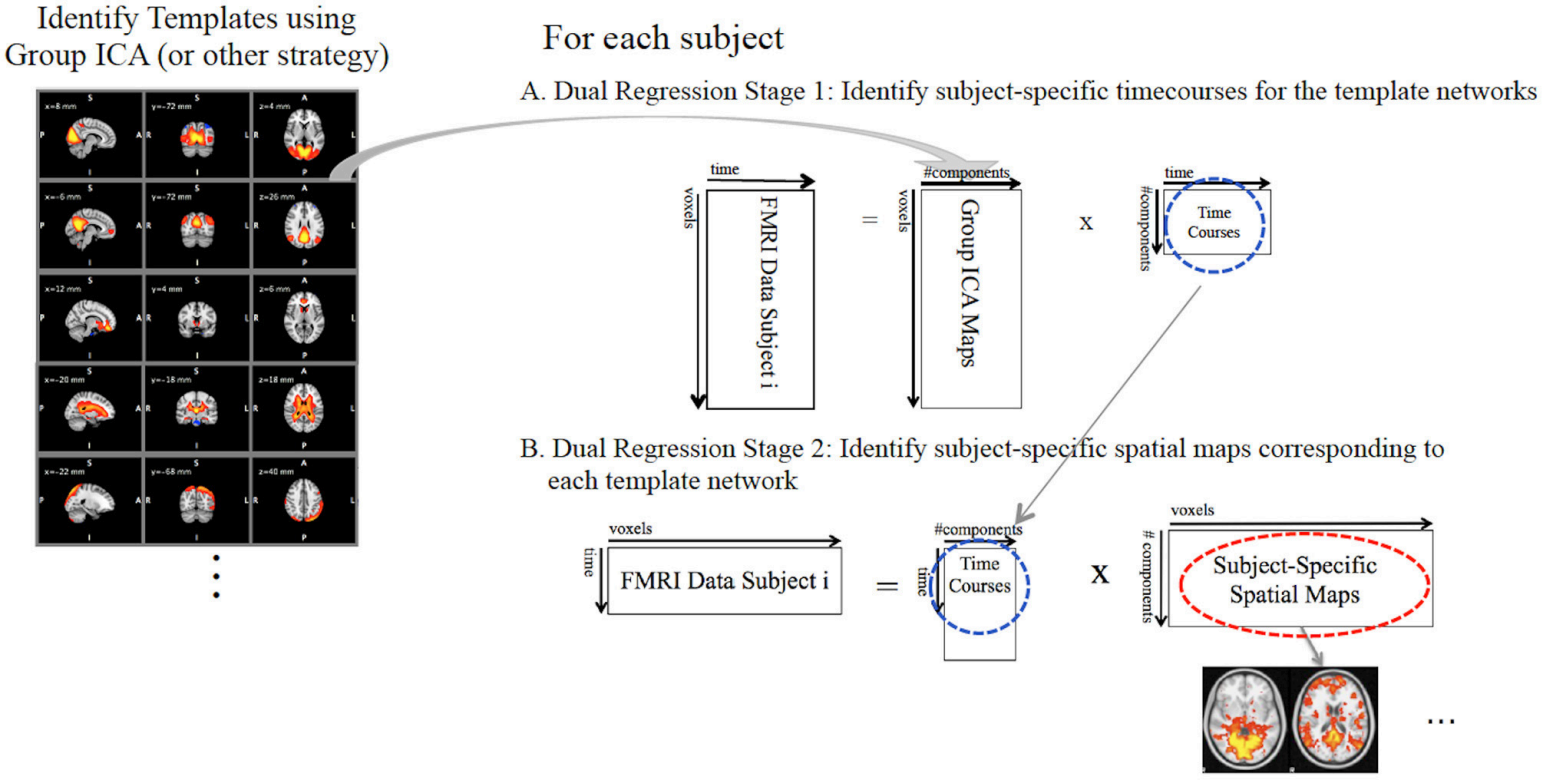
**The dual regression proceeds in two stages**. **(A)** In the first stage, subject-specific timecourses (blue circle) for each template network are extracted using a multivariate spatial regression of the template maps against each subjects FMRI data. **(B)** In the second stage, the subject-specific timecourses from stage 1 are used in a second multivariate regression against the subjects' FMRI data to identify the subject-specific spatial maps (red circle) corresponding to each template network of interest.

Once the template maps (on the basis of GICA or else) have been identified, the dual regression analysis proceeds in two stages (Figure 1). In the first stage of the dual regression (Figure 1A), each subjects' 4D FMRI dataset, Y, is reorganized into a 2D (N voxels × T timepoints) data matrix, and the unthresholded full set of template maps are regressed into this data matrix. e.g., let the template maps be the estimated IC spatial maps from a group ICA, Ŝ, reorganized into a 2D (N voxels × M components) matrix, then these Ŝ are the independent variables or predictors in a multivariate multiple linear regression:

(1) $$\begin{array}{r} \text{Y}=Ŝ{\text{B}}_{TC}+\;{\text{E}}_{1} \end{array}$$

with:

(2) $$\begin{array}{r} {\hat{B}}_{TC}=pinv\left(Ŝ\right)Y \end{array}$$

pinv denotes the matrix pseudoinverse, Y $\in R^{NxT}$ is the subject's FMRI data, $E_{1}\in R^{NxT}$ is the matrix of errors, and ${\hat{B}}_{TC}\in R^{MxT}\;$ is the matrix of stage 1 timecourses, one for each IC map.^1^ Each timecourse in ${\hat{B}}_{TC}$ reflects the average timecourse computed over voxels in the corresponding IC spatial map after taking into account the contributions of the other components to each voxel's timecourse. The regression parameters, ${\hat{B}}_{TC}$, are sensitive to the scale of the maps in Ŝ. However, for dual regression, Ŝ are the same across all subjects.

In the second stage of the dual regression (Figure 1B), the network-specific timecourses from the stage 1 regression are used as predictors in a second multivariate multiple linear regression into the individual subject's FMRI data^2^. This second regression identifies the subject-specific spatial maps, *B*_*SM*_:

(3) $$\begin{array}{r} \text{Y}\prime ={\hat{B}}_{TC}^{\prime }{\text{B}}_{SM}+{\text{E}}_{2} \end{array}$$

Which gives:

(4) $$\begin{array}{r} {\hat{B}}_{SM}=Y*\;pinv\left({\hat{B}}_{TC}\right) \end{array}$$

The estimated ${\hat{B}}_{SM}$ ∈ $R^{NxM}$ have a spatial map for each corresponding IC template map. These maps are the subject's functional connectivity maps for the networks in Ŝ.

Generally, it is the case that RSN timecourses output from stage 1 of the dual regression (Equation 2) will have different amplitudes, measured as the standard deviation of the timecourse, by virtue of these timecourses being average timecourses (weighted sums) computed over the voxels in the RSN (after partialling out other network temporal effects). When these timecourses are regressed into the FMRI data in the second regression (Equation 3), the regression coefficients at each voxel (Equation 4) are estimated with respect to that subject's RSN amplitude or “scale”. Comparing the voxel values in ${\hat{B}}_{SM}$ (e.g., the regression coefficients) across subjects is confounded by this apparent difference in network amplitude across subjects. Note that this is not the case for the scale of the Ŝ since these maps are used for dual regression in all subjects and thus any scaling in the spatial maps will be constant across subjects.

Standardizing the stage 1 timecourses by dividing by the standard deviation of the timecourse prior to estimating Equation 3 obviates the network amplitude (or scale) problem, with regression coefficients that are interpreted as a change in Y per unit standard deviation in X. Subsequently, the voxel-wise regression coefficient for the group analysis will be evaluated in the same units of standard deviation in X across subjects. This is applicable for a network-wide amplitude effect, in which the amplitude of the whole network is affected (e.g., different across subjects). However, another potential problem arises when a region, or node, within a network has an amplitude that is different from the other nodes in the network. In the case of such a “within-network” amplitude effect, a different scale problem arises for the voxels within the network itself—with their regression coefficients being estimated on different scales with respect to each other. Standardizing to change in Y per unit standard deviation in X places all voxels on an equivalent scale, while also placing all subjects on an equivalent scale. As an aside, stage 1 timecourse normalization differs from normalizing the individual voxel's fMRI timecourses prior to the dual regression. In the latter case, even after normalization of the individual voxel-wise timecourses, the subject-specific timecourses from the first stage of the dual regression are still a weighted sum of the data time courses—even if all original time series are zero mean and unit standard deviation, their weighted sum does not remain normalized in either magnitude or variance upon dual regression.

As a consequence, to investigate amplitude effects—or to ensure validity in the case of unknown amplitude effects—the timecourses *must* be normalized by the amplitude prior to estimation of the ${\hat{B}}_{SM}$ using Equation 3, which will give “semi-standardized” regression coefficients, ${\hat{B}}_{SM}^{*}$:

(5) $$\begin{array}{r} {\hat{B}}_{SM}^{*}=Y*pinv\left(A^{-1}*{\hat{B}}_{TC}\right) \end{array}$$

With $A=diag\left\{a_{{\hat{B}}_{TC}}\right\}$, e.g., a diagonal matrix with the standard deviations, or amplitudes a_i_, of each stage 1 timecourse on the diagonal. In FSL dual regression parlance, normalization of these timecourses is referred to as design normalization.

Importantly, ${\hat{B}}_{SM}^{*}$ are sensitive to both network amplitude and shape effects and moreover, are the *only* accurate measure for assessing group differences in functional connectivity in the presence of within-network amplitude effects. See Appendix 1 (Supplementary Material) for a more detailed explanation of this using a simple two-voxel network example.

There are two issues to keep in mind when using ${\hat{B}}_{SM}^{*}$. The first is that there is a price for including the extra amplitude information. The spatial maps fed forward to the group analysis will reflect variations in shape, *and* at the voxel level, the between-subject variations in amplitude. The extra random effects variance from the amplitude will make it more difficult to detect an effect since both effects contribute to the random-effects between-subject variation. The second is that the ${\hat{B}}_{SM}^{*}$ are sensitive to subject motion that manifests as large-amplitude spikes in the timecourses and, as with any RSFC analysis, careful attention must be given to any motion effects in the data. That being said, at least this type of amplitude effect will be accurately localized (which cannot be said for the impact of motion on ${\hat{B}}_{SM}$ or when using SBCA). The fact that motion can mimic amplitude effects likely underlies the overall greater sensitivity of functional connectivity analyses to motion and may be (at least partly) the cause of very unpredictable effects that motion has been shown to have on functional connectivity outcomes. (Satterthwaite et al., 2012; Van Dijk et al., 2012; Power et al., 2014, 2015).

A summary of key points is that:

1. ${\hat{B}}_{SM}$, or the raw regression coefficients of Equation (4), do not contain information related to the network-wide amplitude and thus can't be used to investigate network amplitude.
2. ${\hat{B}}_{SM}$ will be wrong if there is a within-network amplitude difference (e.g., when a node in a network has higher or lower amplitude than other nodes in the network).
3. ${\hat{B}}_{SM}$ also will be wrong in the presence of motion effects that mimic amplitude effects by increasing variance of network timecourses (e.g., motion effects will not be localized only to truly affected voxels).
4. Amplitude information is a source of extra random effects variance.
5. With design normalization, *any* amplitude effect, whether a real network-related effect or one that arises due to motion effects, will be accurately localized in the dual regression stage 2 maps, ${\hat{B}}_{SM}^{*}$.

In this report, we illustrate these concepts using simulated and real data. In the simulations, we manipulate ground truth timecourses and spatial maps derived from real FMRI data to create known group amplitude and shape differences in simulated data. The resulting simulated data are then analyzed using DR to derive ${\hat{B}}_{SM}$ and ${\hat{B}}_{SM}^{*}$ to show how amplitude and shape differences are localized in each. Amplitude effects also arise through impulsive subject movements, thus an analysis of *in vivo* data is used to illustrate the consequences and best practices in using dual regression in the presence of motion.

## Materials and methods

### Simulations: overview

Eight spatial maps corresponding to well-established RSNs (previously reported by Beckmann et al. (2005) and available at http://www.fmrib.ox.ac.uk/analysis/royalsoc8/) were used as ground truth spatial patterns, and timecourses derived from real BOLD FMRI resting state data acquired in 36 healthy subjects were used as ground truth timecourses to construct ground truth resting state FMRI data for 36 simulated subjects that had 8 “active” RSNs (plus noise). Manipulation of derived timecourses and/or ground truth spatial maps in half of the simulated subjects was done to create group differences in functional connectivity.

#### Ground truth spatiotemporal processes

Resting state BOLD FMRI data collected in 36 healthy participants at the University of Oxford Centre for Clinical Magnetic Resonance Research using a 3T Siemens Trio scanner (Filippini et al., 2009) were used for the simulations. The University of Oxford Centre for Clinical Magnetic Resonance Research Ethics Committee approved the original study and all participants gave informed written consent to participate in the original study. Scan parameters were: Field of view = 224 mm, 3 × 3 × 3.5 mm resolution, TE/TR/FA = 28/2000 ms/89°, total scan time = 6:04 min. The first four frames were discarded for magnetic field equilibration. FMRI data were pre-processed using FSL (FMRIB's Software Library, www.fmrib.ox.ac.uk/fsl, Smith et al., 2004) to perform head motion correction using FLIRT (Jenkinson et al., 2002); non-brain removal using BET (Smith, 2002); spatial smoothing by a Gaussian kernal (FWHM 6 mm); grand-mean intensity normalization of the entire 4D dataset by a single multiplicative factor; and highpass temporal filtering by subtraction of a Gaussian-weighted least-squares straight line fitting with sigma = 150 s (Niazy et al., 2011). Finally, registration of each FMRI dataset to the corresponding high-resolution structural scan was carried out using FLIRT. Registration of the high-resolution structural images to MNI152 standard space was achieved using FLIRT, with further refinement using FNIRT non-linear registration (Smith et al., 2004). All subjects' four-dimensional timeseries data were transformed into standard space at 2 × 2 × 2 mm^3^ resolution using the registration transformation matrices.

Ground truth spatial maps: The spatial network maps from Beckmann et al. (2005) were used as the ground truth spatial maps, hereafter referred to as the roysoc8 maps. These eight maps correspond to (A) medial visual network (MVN), (B) lateral occipital, (C) auditory, (D) sensorimotor, (E) default mode network (DMN), (F) executive control network (ECN), and (G–H) right- and left-lateralized fronto-parietal networks (RFPN and LFPN, respectively).

Ground truth network timecourses: The roysoc8 maps were regressed against one of the real pre-processed FMRI datasets to derive 8 corresponding “ground truth” RSN timecourses per subject. The voxel-wise standard deviations estimated from the residuals of the regression (*std*_*res*_) for each subject were also calculated.

#### Simulated resting state FMRI data

Simulated single subject resting state FMRI data were created by computing the product of the roysoc8 maps and one set of ground truth RSN timecourses, and adding (at each time point) voxel-wise random Gaussian noise distributed as $N\left(0,std_{res}^{2}I\right)$ plus the voxel-wise means of the real FMRI timeseries. Simulated data created from half of the real FMRI data (18 subjects) were designated as Group A data, the other half (18 subjects) as Group B.

**Figure 2:**
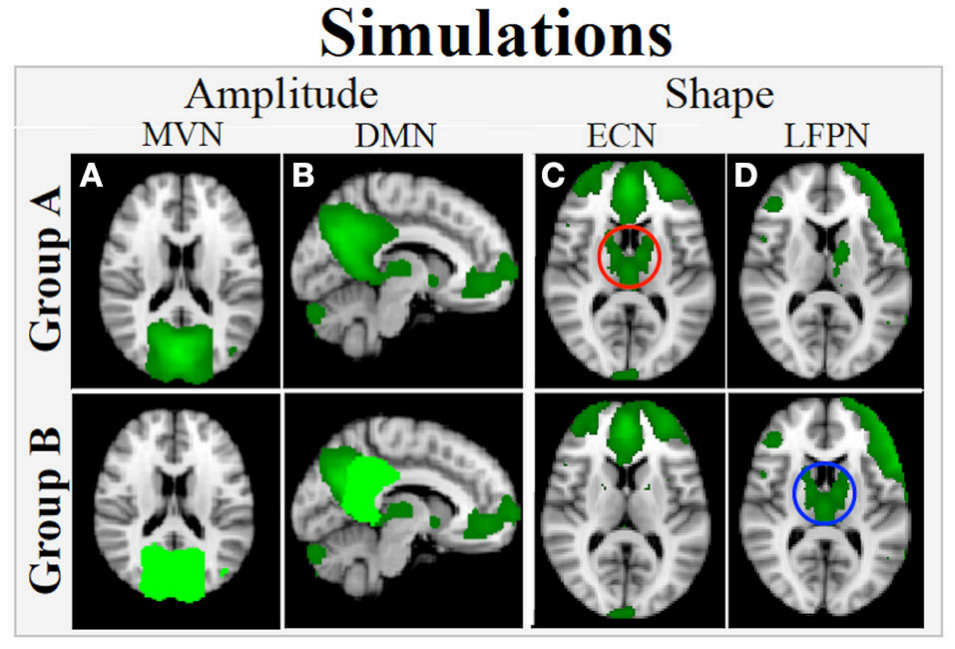
**Simulated between-group amplitude and shape differences**. **(A)** Network-wide amplitude effect in MVN: Voxels in the entire network are assigned a ground truth timecourse for each subject in Group A (upper panel; dark green) and 1.1^*^ground truth timecourse for those in Group B (lower panel; bright green). **(B)** Within-network amplitude effect: For subjects in Group A, all voxels in the network are assigned a network timecourse (upper panel; dark green), but for Group B, network voxels in dark green are assigned the ground truth timecourse and voxels in bright green are assigned 1.5^*^ ground truth timecourse (lower panel, **C,D**) Network shape difference: in Group A, basal ganglia and thalamus are connected with ECN (**C**, upper panel, red circle) and in Group B, this region is connected with LFPN (**D**, lower panel, blue circle).

For Group B data, manipulation of the timecourses and/or associated spatial maps prior to multiplication of timecourses with the spatial maps was done to simulate between-subject variability in RSN amplitude and shape as follows^3^:

- Network-Wide Amplitude Difference (Figure 2A): For each subject, the ground truth timecourse for the MVN was multiplied by 1.1 to simulate a 10% greater network-wide amplitude in Group B relative to Group A.
- Within-Network Amplitude Difference (Figure 2B): For each subject, the ground truth timecourses from voxels in posterior cingulate cortex (PCC) region of the DMN were multiplied by 1.5 to simulate a 50% greater amplitude of the PCC relative to other regions in the DMN. This enforces a within-network group amplitude difference, e.g., a within-network difference around the mean network amplitude.
- Shape (Figures 2C,D): Voxels in selected regions of the ECN (basal ganglia and thalamus) were “connected” to the LFPN in Group B subjects by giving that subset of voxels the timecourse for LFPN instead of the timecourse for the ECN. The net effect is that the basal ganglia and thalamus are connected to ECN in Group A (Figure 2C) and to LFPN in Group B (Figure 2D), resulting in a group difference in shape in both ECN and LFPN. Note that the timecourses used here where not normalized to unit standard deviation and thus there will also be a small amplitude difference in the overall network timecourses.

Note that each amplitude and shape effect impacts a single map when assessing differences in functional connectivity between Group A and Group B, with a network-wide amplitude difference in MVN, a within-network difference in DMN, and shape differences in ECN and LFPN. The other maps did not have any simulated group differences. All effects were included in a single simulated Group B dataset to also show that dual regression can separate out multiple different network amplitude and shape effects that may be present in any given FMRI dataset.

#### Group comparisons of RSN functional connectivity using dual regression

Dual regression (http://fsl.fmrib.ox.ac.uk/fsl/fslwiki/DualRegression) was done as follows. At stage 1, the roysoc8 spatial maps were regressed against the simulated FMRI datasets to obtain the subject-specific timecourses (SSTC). The SSTC outputs are text files, one per subject, containing a matrix of values that have one column per spatial map and one row per time point.

At stage 2 of the dual regression, the SSTC (for all networks) were regressed against each of the simulated datasets to obtain the subject-specific spatial maps (SSSM). Each stage 2 ouput is a 4D spatial maps data file per subject (*N* = 8 components in this case). Dual regression was run without and with normalization of the SSTC (each timecourse normalized to unit standard deviation), resulting in SSSM that were ${\hat{B}}_{SM}$ or ${\hat{B}}_{SM}^{*}$, respectively.

Once the ${\hat{B}}_{SM}$ and ${\hat{B}}_{SM}^{*}$ were computed, estimation and inference on the two different sets of statistic maps was done using FSL Randomise with a two-group unpaired *t*-test to assess differences between Group A and B in each network and for each statistic (${\hat{B}}_{SM}$ and ${\hat{B}}_{SM}^{*}$), with 5,000 permutations and cluster-mass based thresholding (with cluster forming threshold Z = 2.3), to achieve a corrected family-wise error rate, *p* < 0.05.

SSTCs are evaluated for accuracy by comparing the SSTC with the ground truth timecourses. The correlation between each ground truth timecourse and the corresponding dual regression timecourse for every network should be close to 1. We also compared the amplitudes (standard deviations) of the ground truth timecourses with the amplitudes of the SSTC for the MVN (the component with the 10% amplitude effect) and the other networks. For MVN, the amplitudes of SSTC and ground truth timecourses should be equal in Group A, and for Group B, the amplitudes of the SSTC should be equal to 1.1 times the amplitudes of the ground truth timecourse for each subject. There will also be an amplitude effect in the DMN because the SSTC is a weighted sum of all voxel's timecourses in the DMN and some voxels have a larger amplitude than others, although this is not easily estimated as is the network-wide effect. For the other five networks, SSTCs should be the same as the ground truth timecourses.

The results of the group-level analysis of the ${\hat{B}}_{SM}$ and ${\hat{B}}_{SM}^{*}$ spatial maps should show group differences in MVN, DMN, ECN, and LFPN that reflect the voxel-wise differences in amplitude/synchrony as shown in Figure 2, with no differences in functional connectivity in the other four networks.

### *In Vivo* resting state FMRI: overview

An *in vivo* resting state FMRI dataset from 13 healthy controls (HC), 14 individuals with bipolar disorder (BD), and 12 individuals with schizophrenia (SZ), for a total of 39 subjects, is used to illustrate how motion can distort functional connectivity results. Analyses to assess differences in functional connectivity between the three diagnostic categories was done two ways: (1) using all of the data (*N* = 39), which included several subjects in each group with motion in excess of 1–2 voxels, and (2) using a subset of the data that included only subjects with less than 1.5 mm motion (*N* = 25). These two analyses demonstrate how excess motion can impact GICA maps and group differences in functional connectivity and how using strict motion criteria can improve findings even at the expense of sample size. In addition, we ran a group analysis that compared “high” vs. “low” motion subjects (with these two groups matched for the same number of participants from each diagnostic category) to show how motion can cause spurious group differences in functional connectivity.

As an aside, we are not interested in the neurobiological differences between individuals with different diagnoses *per se* and will not be discussing our findings in this context; details of the participants and neurobiological research findings have been reported previously (Öngür et al., 2010; Chai et al., 2011).

#### Data acquisition

MRI data were collected at the McLean Imaging Center at McLean Hospital using a Siemens Trio 3T scanner. The local Institutional Review Board of McLean Hospital approved the original study and all participants in the original study gave written informed consent. High-resolution anatomical images were acquired for registration purposes using an MPRAGE sequence with 256 × 256, 1 × 1.3 mm in-plane resolution, and 1.3 mm slice thickness. Resting state BOLD FMRI data were acquired while subjects rested quietly with eyes open. Scan parameters were: 42 slices, 3.5 × 3.5 × 3.5 mm resolution, TE/TR/FA = 24/2,500 ms/82°, interleaved slice acquisition, 64 × 64 matrix, total scan time = 10 min. The first four frames were discarded for magnetic field equilibration.

#### Pre-processing

Data pre-processing was done similarly to the simulated data and included: Head motion correction using FLIRT; non-brain removal using BET; spatial smoothing by a Gaussian kernal (FWHM 5 mm); grand-mean intensity normalization of the entire 4D dataset by a single multiplicative factor; and highpass temporal filtering by subtraction of a Gaussian-weighted least-squares straight line fitting with sigma = 150 s. Finally, registration of each subject's FMRI data to their high-resolution structural scan was carried out using FLIRT. Registration of the high-resolution structural images to MNI152 standard space was achieved using FLIRT, with further refinement using FNIRT non-linear registration. All subjects' four-dimensional timeseries data were transformed into standard space at 2 × 2 × 2 mm^3^ resolution using the registration transformation matrices.

#### GICA-DR

**Figure 3:**
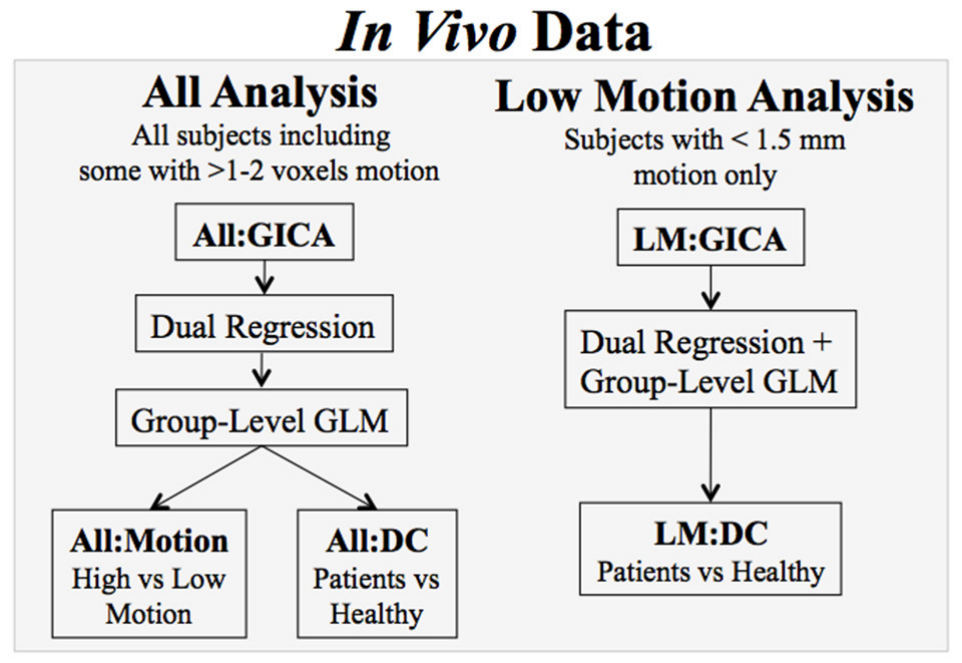
**Analyses of the ***in vivo*** data**. Two different GICA-DR analyses were done, one that included all subjects, regardless of level of motion (All), and a second that included participants with <1.5 mm motion in any direction (Low Motion). Different group level models were conducted to assess motion effects, e.g., with participants grouped either by level of motion or by diagnostic category (DC).

Figure 3 shows an illustration of the analyses.

**All Analysis:** For this analysis, all participants' data (*N* = 39) were included in the All:GICA, dual regression, and group-level models, regardless of level of motion. In the **All:Motion** group-level analysis, a two group GLM was set up to compare functional connectivity between two artificially constructed groups based on participant's absolute mean displacement being either smaller than 1 mm (“low motion”; *N* = 20) or larger than 1 mm (“high motion”; *N* = 19), irrespective of diagnostic category. With 1 mm as the cutoff, nearly equal numbers of participants from each diagnostic category are included in each group (*N* = 7 HC in both, *N* = 6 SZ in both, *N* = 7 BPD in “low” and *N* = 6 BPD in “high”), thus diagnostic category was matched across the two groups. In the **All:DC** group-level analysis, a diagnostic category three-group GLM was done to compare functional connectivity between the patient groups and the healthy controls. This analysis included some participants with high levels of motion in each group.

**Low Motion (LM) Analysis:** This analysis used a stricter motion criteria, removing all participants with >1.5 mm motion prior to the LM:GICA and subsequent dual regression and group-level GLM (leaving 6 BD, 10 HC, and 9 SZ). An additional HC participant was also excluded from this analysis even though the max absolute mean displacement was <1 mm because a rotational movement of 0.006 radians around the z axis by the subject led to a large signal change in the dual regression timecourses (this is further discussed in the results section). A single group GLM was done, **LM:DC**, which was a diagnostic category-based three-group GLM to assess group differences between patient groups and the healthy controls. This analysis did not include any participants with high levels of motion and can thus be compared with All:DC to assess how motion can corrupt functional connectivity results. For All:DC and LM:DC, we use results for HC vs. SZ which have approximately the same number of subjects in each group for both analyses.

**All:GICA and LM:GICA**: The corresponding FMRI data for the subjects included in each GICA (*N* = 39 for All:GICA and *N* = 25 for LM:GICA) were concatenated together in the temporal dimension and the group ICAs were done using FSL MELODIC (Multivariate Exploratory Linear Decomposition into Independent Components) Version 3.09 (Beckmann and Smith, 2004; Beckmann et al., 2005). The number of components was fixed to twenty for both analyses to give maps similar to those reported in Beckmann et al. (2005). To obtain a stable decomposition of the data, each GICA was run eight times followed by a meta-level GICA fed by all of the spatial maps from the 8 decompositions (Smith et al., 2009; Wisner et al., 2013). The meta-level analysis for the All:GICA estimated 17 components and for the LM:GICA estimated 20 components.

**Dual Regression and Group-Level Statistical Analysis:** The dual regressions for the All and Low Motion analyses were implemented as described in the simulations, to obtain the statistic maps (${\hat{B}}_{SM}$, ${\hat{B}}_{SM}^{*}$) for each RSN for each subject for each analysis. Estimation and inference on group differences in functional connectivity using ${\hat{B}}_{SM}$ and ${\hat{B}}_{SM}^{*}$ was done using FSL Randomise (5,000 permutations, cluster-based thresholding, Z = 2.3 cluster forming threshold) for each network at *p* < 0.05, corrected.

## Results of simulations: group differences in RSN shape and amplitude

### Accuracy of stage 1 timecourses

**Figure 4:**
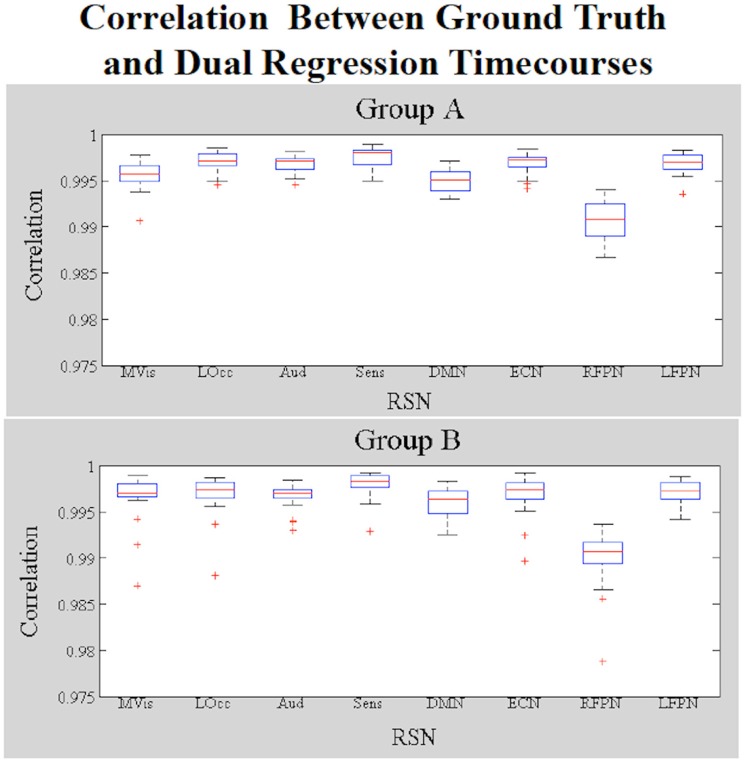
**Simulation Results**. Boxplots of the correlations between the stage 1 and ground truth timecourses for each network (19 subjects in each group). Data in Group B had amplitude and shape differences in some networks.

**Figure 5:**
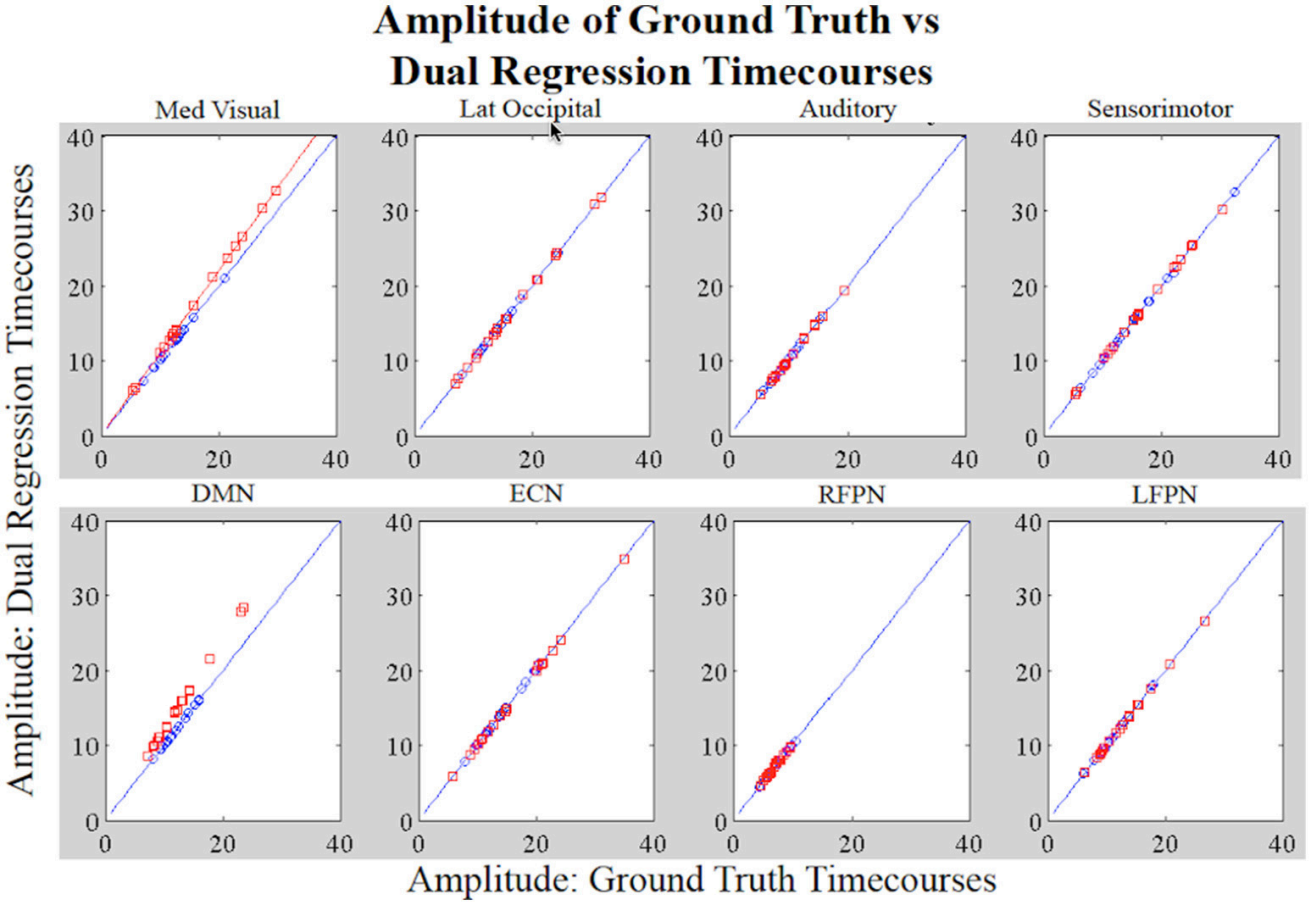
**Simulation Results**. Each datapoint shown in the plots is the amplitude of the original RSN timecourse vs. the amplitude of the stage 1 timecourse for the subject assigned that timecourse (Group A: blue; Group B: red). For MVN, the network-wide amplitude difference between Groups A and B is reflected by the different slopes (Group A: *y* = *x* and Group B: *y* = 1.1^*^x; *x* = original timecourses). For the DMN, the within-network amplitude differences between groups are also reflected by datapoints in Group B not laying on the same line as those in A. All other networks show no group differences in amplitude.

Figure 4 shows a boxplot of the correlation coefficients between each stage 1 timecourse and each ground truth timecourse for each network for subjects in Groups A and B. We did not alter the shape of the temporal trace for any of the RSNs in the simulated data and the dual regression is able to faithfully reproduce the temporal response of the ground truth timecourses, with the correlations between stage 1 timecourses and ground truth timecourses all near 1. Figure 5 shows the amplitudes of the stage 1 timecourses are as expected, with: A 10% difference in network amplitude in the MVN, reflected in the slopes of the lines of best fit for Group A and Group B, a difference in amplitude (e.g., slope) between the two groups in the DMN, and no difference in amplitudes for any of the other networks.

#### Group differences in RSN functional connectivity: amplitude effects

**Figure 6:**
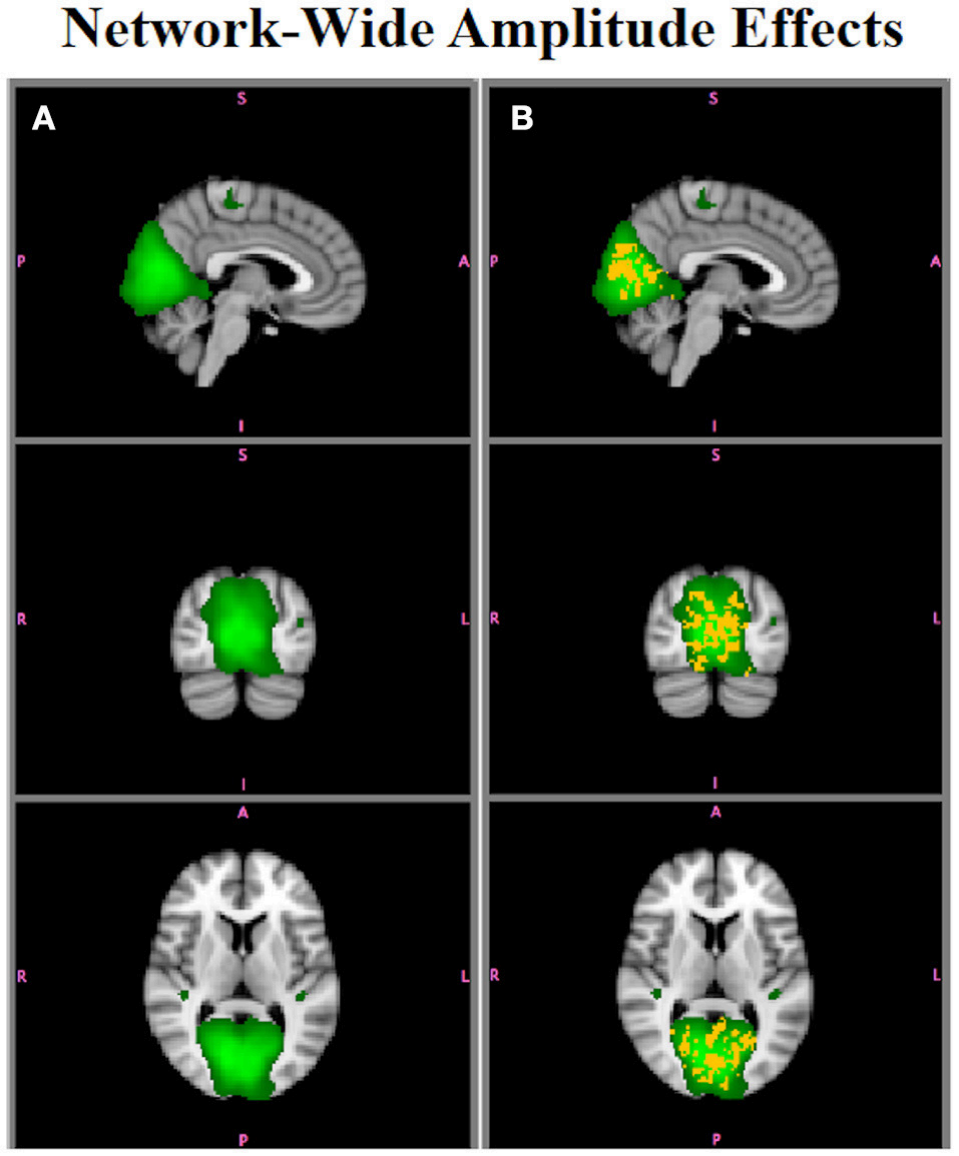
**Simulation Results**. **(A)** Comparison of functional connectivity between Group A and Group B using ${\hat{B}}_{SM}$ does not show any group differences in the MVN (e.g., ${\hat{B}}_{SM}$ are not sensitive to within-network amplitude effects). **(B)** ${\hat{B}}_{SM}^{*}$ display within-network amplitude effects (red-yellow, B > A, *p* < 0.05).

Figure 6 shows differences in functional connectivity between Group A and Group B for the MVN, which had simulated network-wide amplitude differences. In this and all subsequent brain image figures, three orthogonal slices are displayed in each vertical panel, with the roysoc8 RSN spatial map shown in green overlaid onto the MNI 2 mm standard brain image. Statistically significant positive differences are shown in red-yellow and negative differences are shown in blue-lightblue. All results are significant at *p* < 0.05 corrected. Figure 6A shows differences assessed using ${\hat{B}}_{SM}$ as inputs to Randomize, and Figure 6B shows those using ${\hat{B}}_{SM}^{*}$. ${\hat{B}}_{SM}$ are not sensitive to network-wide amplitude effects, whereas ${\hat{B}}_{SM}^{*}$ are sensitive to network-wide amplitude differences, with the voxels showing significant differences lying only within the network that were simulated to have an amplitude effect (Figure 2A).

**Figure 7:**
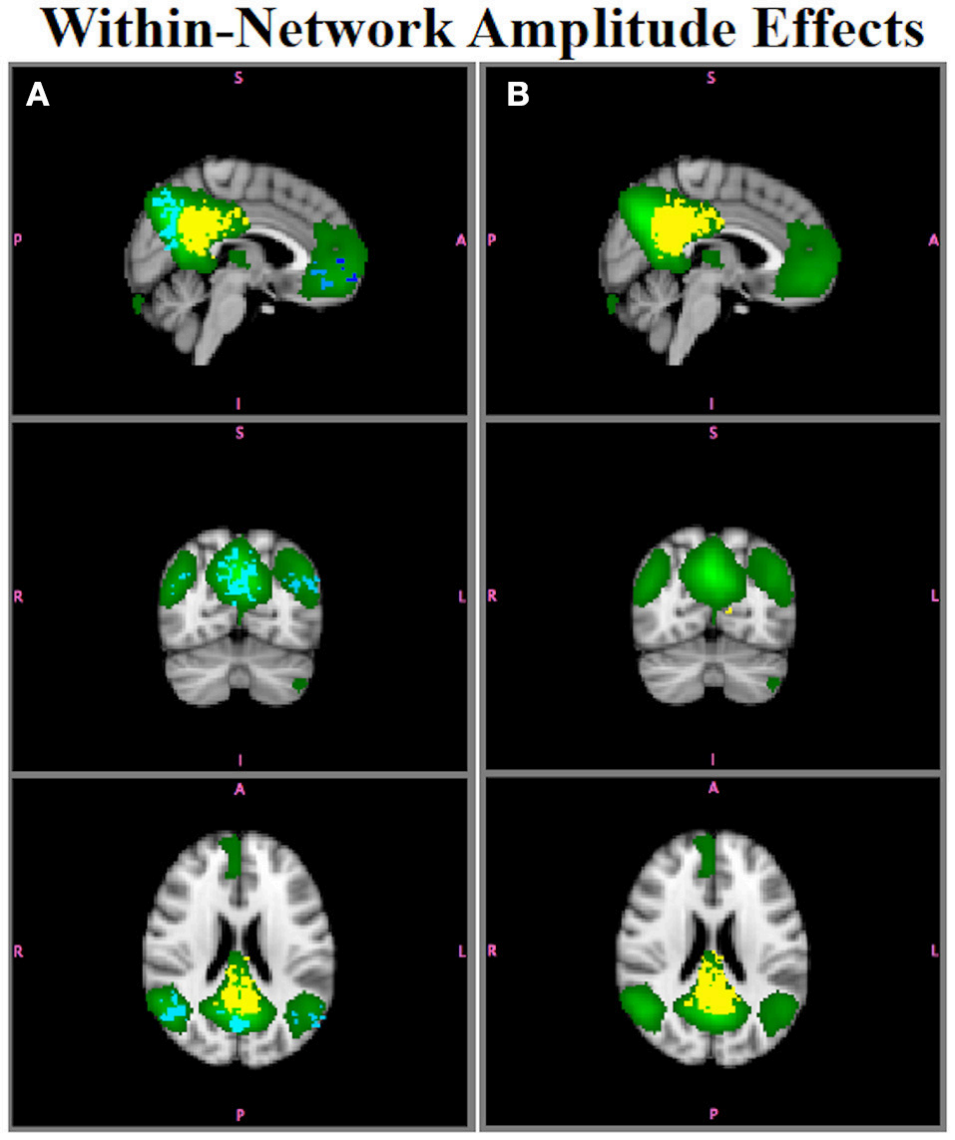
**Simulation Results**. The DMN (green) had a within-network amplitude difference in Group B, but not in Group A, in the posterior cingulate ROI. **(A)** ${\hat{B}}_{SM}$ shows group differences in connectivity throughout the network (yellow, B > A; blue, A > B (e.g., false positives); *p* < 0.05. **(B)** ${\hat{B}}_{SM}^{*}$ accurately localize the group differences only to posterior cingulate (yellow).

Figure 7 shows results for comparison of Group A and Group B for the DMN. Figure 7A shows group differences assessed using ${\hat{B}}_{SM}$ as inputs to Randomize, Figure 7B shows those using ${\hat{B}}_{SM}^{*}$. Although the PCC region was the only region with an amplitude difference between Groups A and B in the DMN (Figure 2B), using ${\hat{B}}_{SM}$ would lead one to conclude that there were group differences throughout the entire network, with some regions showing greater connectivity in Group B relative to Group A (red-yellow) and others showing decreased connectivity in Group B relative to Group A (blue-light blue). $\;{\hat{B}}_{SM}^{*}$ accurately localizes within-network amplitude differences to only the PCC region with the correct sign (e.g., B > A).

#### Group differences in RSN shape

**Figure 8:**
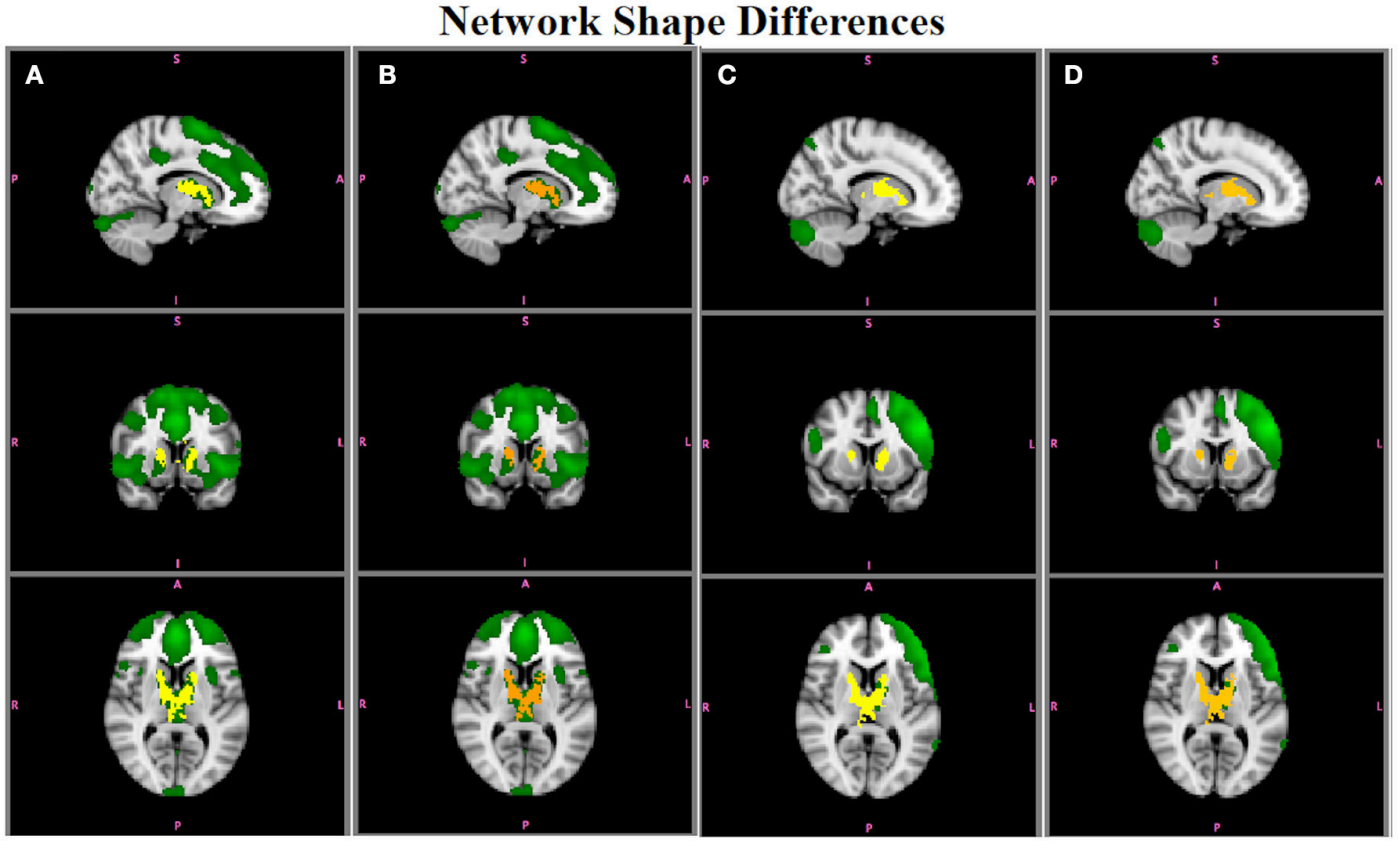
**Simulation Results**. Group differences in the shape of the ECN obtained using **(A)** ${\hat{B}}_{SM}$ and **(B)** ${\hat{B}}_{SM}^{*}$ (red-yellow; A > B; *p* < 0.05), and in the LFPN using **(C)** ${\hat{B}}_{SM}$ and **(D)** ${\hat{B}}_{SM}^{*}$ (red-yellow; B > A). Both statistics accurately reflect the group difference in shape in ECN and LFPN in the absence of amplitude effects.

Figures 8 show the results for RSNs with simulated shape differences (shown in Figures 2C,D). Both statistics are capable of accurately reflecting the differences in functional connectivity when those differences are due only to shape differences (or synchrony between the timecourses from different areas in the network) and not amplitude. In Figures 8A,B, the ECN is more strongly connected to caudate/thalamus regions in Group A, using either ${\hat{B}}_{SM}^{\;}$ or ${\hat{B}}_{SM}^{*}$, respectively. Figures 8C,D show that the LFPN is more strongly connected with caudate/thalamas in Group B, with ${\hat{B}}_{SM}^{\;}$ and ${\hat{B}}_{SM}^{*}$, respectively. In both cases, ${\hat{B}}_{SM}^{*}$ have a slightly lower *p*-values than ${\hat{B}}_{SM}^{\;}$ (e.g., brighter yellow colors indicate more significant differences than orange colors) because of the extra random effects variation due to including amplitude information in ${\hat{B}}_{SM}^{*}$, but not in ${\hat{B}}_{SM}^{\;}$. No other networks showed group differences in shape.

### Results of the *in vivo* analysis: motion and functional connectivity

#### All:GICA vs. LM:GICA

**Figure 9:**
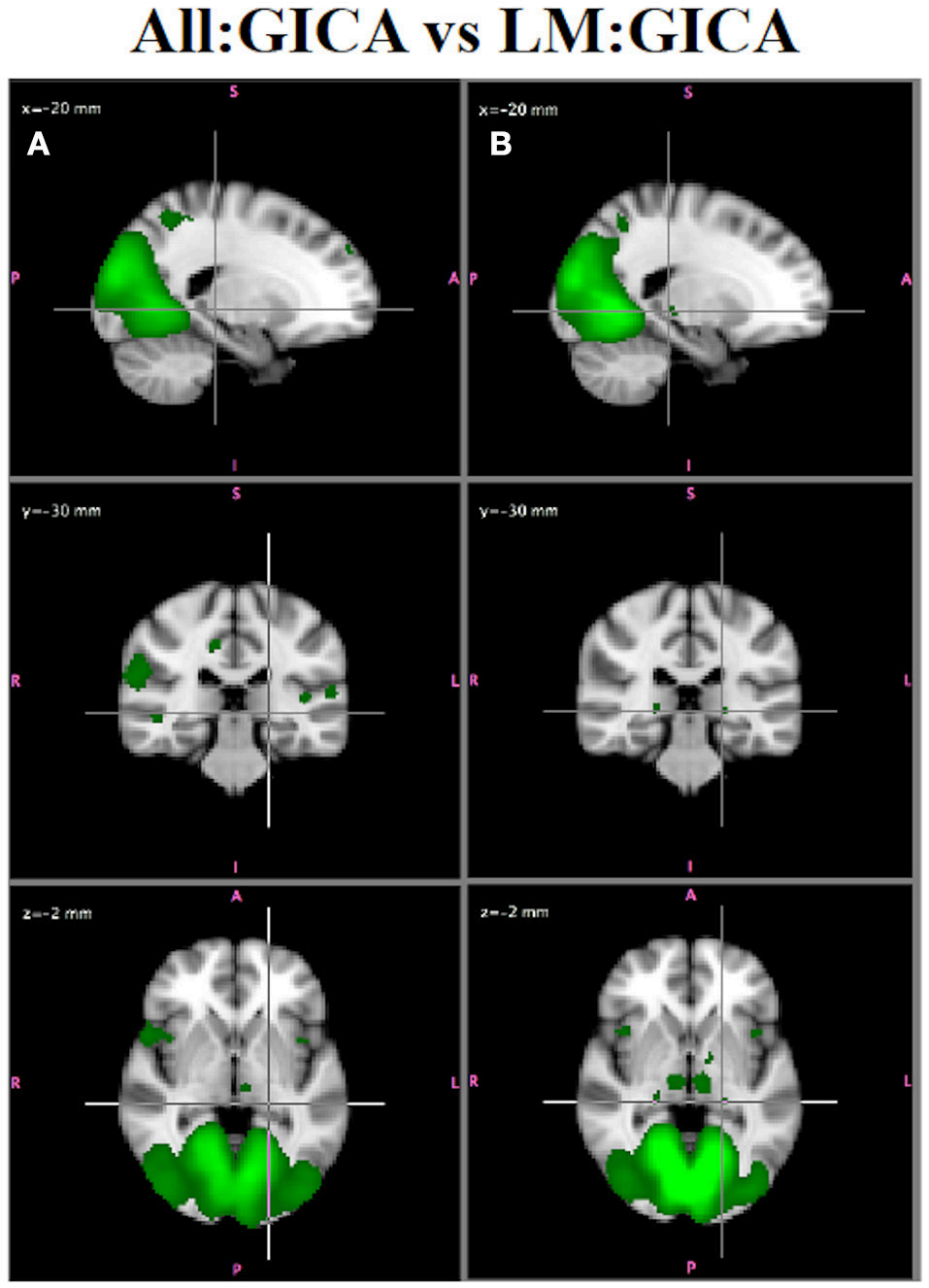
*****In Vivo*** Results**. MVN from: **(A)** All:GICA (green, Z > 2.3) and **(B)** LM:GICA. The LM:GICA map also includes lateral geniculate nucleus (crosshair) and bilateral thalamus, which are not observed in the All:GICA MVN.

Visual inspection of the independent component spatial maps shows that removing participants with greater motion generally resulted in improvements in the spatial maps, although the GICA maps from both analyses highly resembled (via spatial cross correlation) the roysoc8 maps for 7/8 major networks (all but LFPN, which showed some variability). This is not surprising given that ICA is effective for denoising FMRI data by identifying and separating out effects due to both physiological and subject motion (McKeown et al., 1998; Kiviniemi et al., 2003; Beckmann et al., 2005; Salimi-Khorshidi et al., 2014; Du et al., 2016). However, there were some subtle differences in the two sets of maps that demonstrated that the LM:GICA generally produced the more reasonable maps. For example, Figure 9 shows the MVN from the All:GICA (Figure 9A) and from the LM:GICA (Figure 9B). While the MVNs identified from both analyses do largely resemble each other, in the LM:GICA map, smaller bilateral regions in the lateral geniculate nucleus and the thalamus are identified in the MVN (Figure 9B) that are not seen in the All:GICA map (Figure 9A). The presence of these regions in MVN is consistent with known connections of LGN and thalamus with visual cortex (Castelo-Branco et al., 1998; Kwon and Jang, 2014), indicating the Low Motion MVN is more reasonable. The LGN were observed in the All:GICA map at a lower threshold of Z = 1.63, but this threshold also showed many other widespread regions (although we did not directly compare these two maps for statistically significant differences).

#### Spurious group differences in RSN functional connectivity due to motion

**Figure 10:**
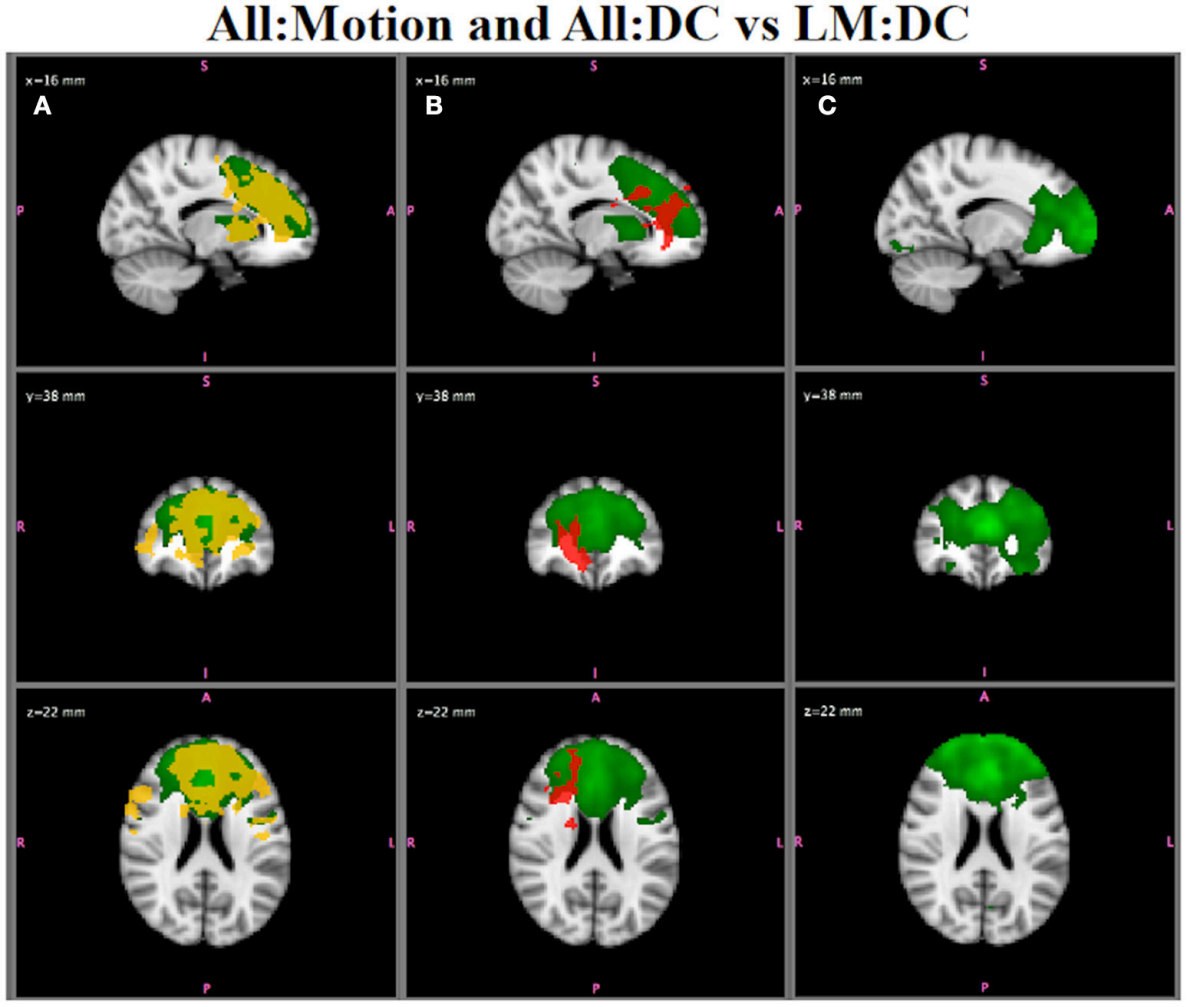
*****In Vivo*** Results**. **(A)** All:Motion analysis: the ECN (green) shows statistically significant differences in functional connectivity for high motion > low motion (red-yellow; *p* < 0.05). **(B)** All:DC analysis: the ECN shows group differences for SZ > HC (*p* < 0.05) in regions overlapping with the motion effects in **(A)**. **(C)** Low Motion analysis: group differences in ECN between SZ and HC are no longer observed, even at very low statistical thresholds. Note that the IC map shown in A and B is from the All:GICA. The IC maps shown in C is from the LM:GICA.

The All:Motion analysis, which compared RSN functional connectivity in individuals with <1 mm motion vs. those with >1 mm motion, showed several networks with spurious group differences in functional connectivity, for inference on both ${\hat{B}}_{SM}^{*}$ and ${\hat{B}}_{SM}^{\;}$. For example, Figure 10A shows that the ECN (green) has widespread group differences in functional connectivity (red-yellow; using ${\hat{B}}_{SM}^{*}$). Since the two groups being compared are matched for diagnostic category, these spurious group differences are likely due to the effects of motion. Inspection of the results for All:DC (Figure 10B; SZ > HC) also shows differences in functional connectivity in this network in regions that overlap the map in Figure 10A, suggesting that motion is affecting the clinical group comparison as well. Indeed, if a strict motion criteria is applied as in the LM:DC analysis (Figure 10C), the group differences in functional connectivity between SZ and HC seen in Figure 10B are no longer observed, even at very low thresholds. Although we did attempt to keep the number of subjects in each analysis comparable, there were fewer subjects in the LM:DC analysis (9HC/9SZ) than in the All:DC analysis (14HC/12SZ), which could also reduce the ability to detect group differences, although lowering the statistical threshold still did not show any group differences in LM:DC.

**Figure 11:**
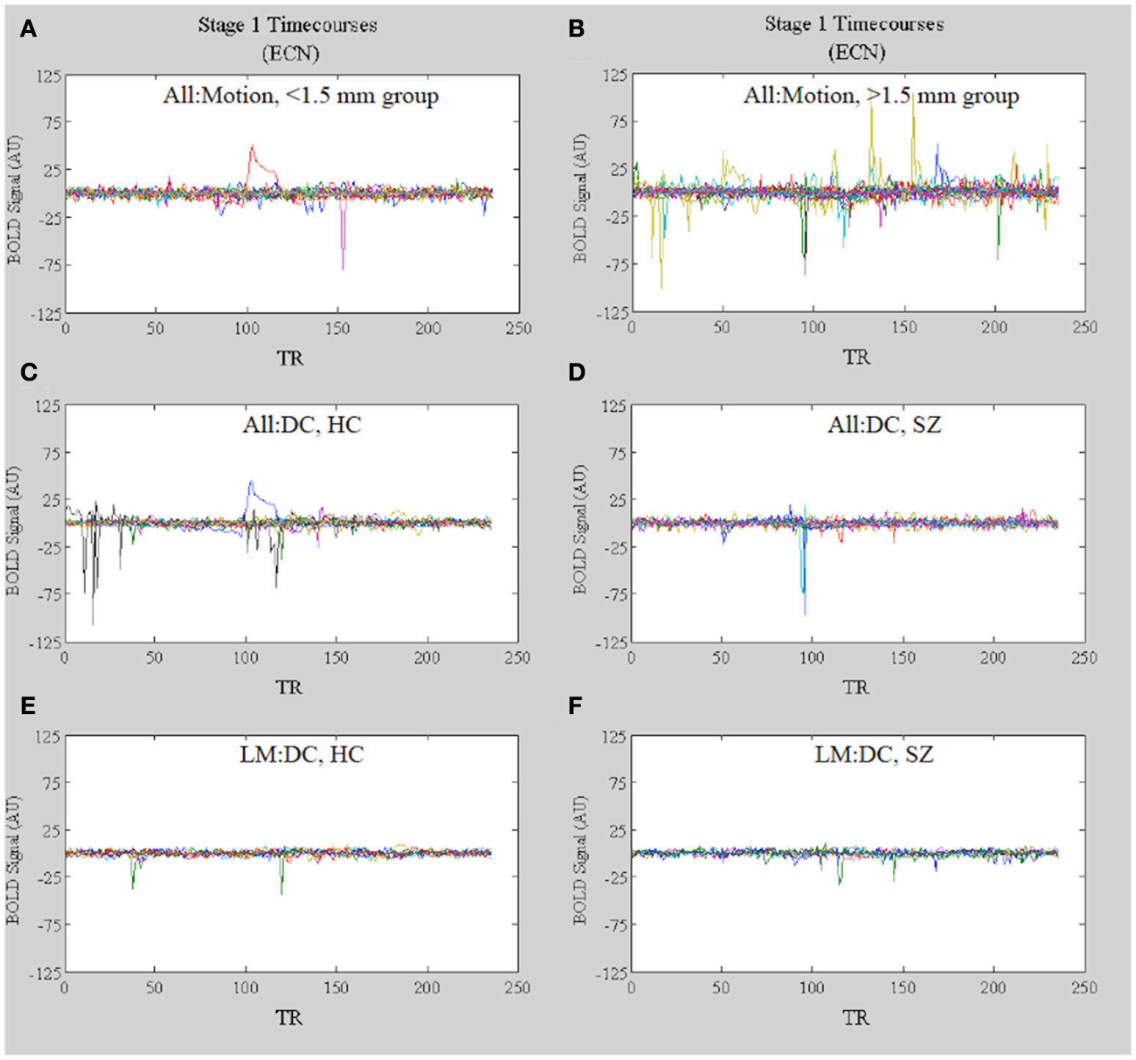
*****In Vivo*** Results**. Stage 1 timecourses for the ECN for: **(A)** All:Motion, lowest motion subjects (<1.5 mm) and **(B)** All:Motion, highest motion subjects (>1.5 mm); **(C)** HC included in the All:DC analysis and **(D)** SZ included in the All:DC analysis; **(E)** HC included in the LM:DC analysis and **(F)** SZ included in the LM:DC analysis. Note that the timecourses for the bipolar patients are included in B but not in **(D)** or **(F)**.

Further evidence that motion-related amplitude effects underlie the observed differences in network functional connectivity observed in the All:Motion and All: DC analyses (Figures 10A,B, respectively) comes from inspection of the dual regression network timecourses for each subject in the high (>1.5 mm) vs. low (<1.5 mm) motion groups. The stage 1 timecourses (Figure 11A vs. Figure 11B) clearly show that many subjects in the high motion group have greater amplitudes (or standard deviations) in the network timecourses. Inspection of the stage 1 timecourses for SZ and HC included in the All:DC vs. LM:DC analyses (Figures 11C,D) shows that there were several HC and SZ with spikes and other motion-related temporal features (identified by comparison with the motion timecourses) included in All:DC that were removed for LM:DC. Another point of note is that the timecourse with the large wide deviation over several TRs in panels Figure 11A (red line) and Figure 11C (blue line) closely resembles the timecourse of the McFLIRT estimated z-rotation for that subject (*r* = −0.76), however the estimated rotation was less than 0.006 radians, which did not result in any appreciable effect in the mean displacement (not shown). Inspection of the raw FMRI data for this subject clearly showed the rotation and the motion correction was not able to appreciably fix it. This suggests that the dual regression procedure can be sensitive to small levels of motion and that inspection of the stage 1 timecourses can provide key information as to whether or not motion may be corrupting functional connectivity results.

#### Loss of sensitivity to group differences in RSN functional connectivity due to motion

**Figure 12:**
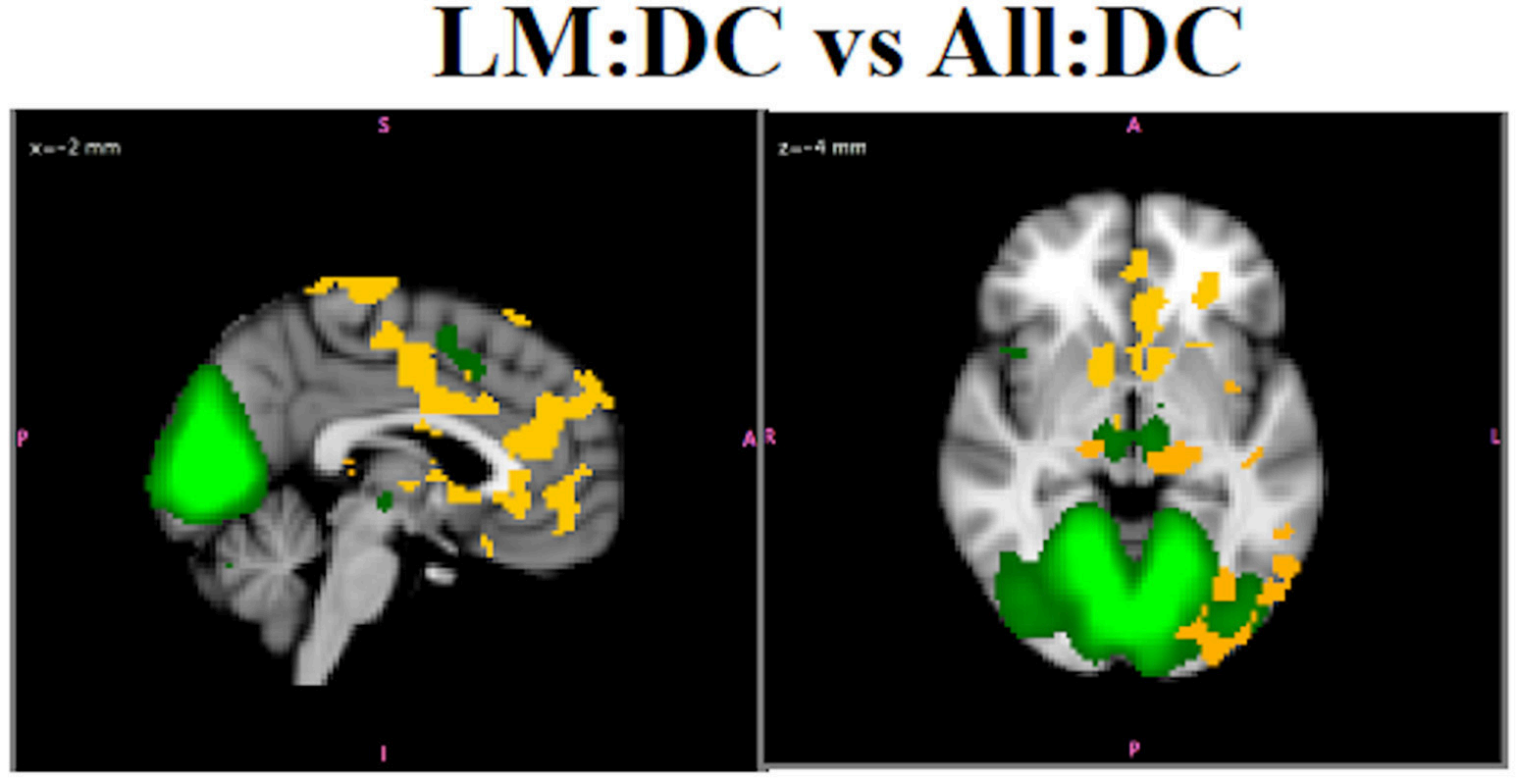
*****In Vivo*** Results**. Results. LM:DC showed differences in the connectivity of MVN (green), with SZ > HC, in lateral and medial geniculate nuclei, bilateral thalamus, nucleus accumbens, insula, regions in PFC, and ACC, and precuneus (red-yellow, *p* < 0.05).There were no group differences for HC vs. SZ in this network for the ALL:DC analysis.

Including participants with higher levels of motion (>1.5 mm) in All:DC obscured functional connectivity differences between SZ and HC in MVN that were revealed in the LM:DC with these subjects removed. Figure 12 shows the differences in functional connectivity for SZ > HC (*p* < 0.05 corrected) using ${\hat{B}}_{SM}^{*}$ for LM:DC. No statistically significant group differences in this network were observed in the All:DC. For LM:DC, group differences using ${\hat{B}}_{SM}$ (not shown) were observed in some regions that overlapped with ${\hat{B}}_{SM}^{*}$. However, there were also differences in white matter and brainstem that were not observed with ${\hat{B}}_{SM}^{*}$, and some of the bilateral differences, for example, in the nucleus accumbens, that were observed using ${\hat{B}}_{SM}^{*}$ were not observed in the ${\hat{B}}_{SM}$ results, underscoring the fact that inference on ${\hat{B}}_{SM}^{*}$ and ${\hat{B}}_{SM}$ will generally not give the same results. Since ${\hat{B}}_{SM}^{*}$ are able to accurately localize any amplitude effects, whereas ${\hat{B}}_{SM}$ will mislocalize any within-network amplitude effects, it is recommended to only infer on ${\hat{B}}_{SM}^{*}$ maps even though amplitude and synchronicity effects cannot be disambiguated without conducting additional analyses. Notably, the group differences in bilateral LGN and nucleus accumbens, that were observed using ${\hat{B}}_{SM}^{*}$ are in agreement with what is know about visual disturbances in schizophrenia (Silverstein and Keane, 2011; Yoon et al., 2013), providing additional support that ${\hat{B}}_{SM}^{*}$ are most valid.

**Figure 13:**
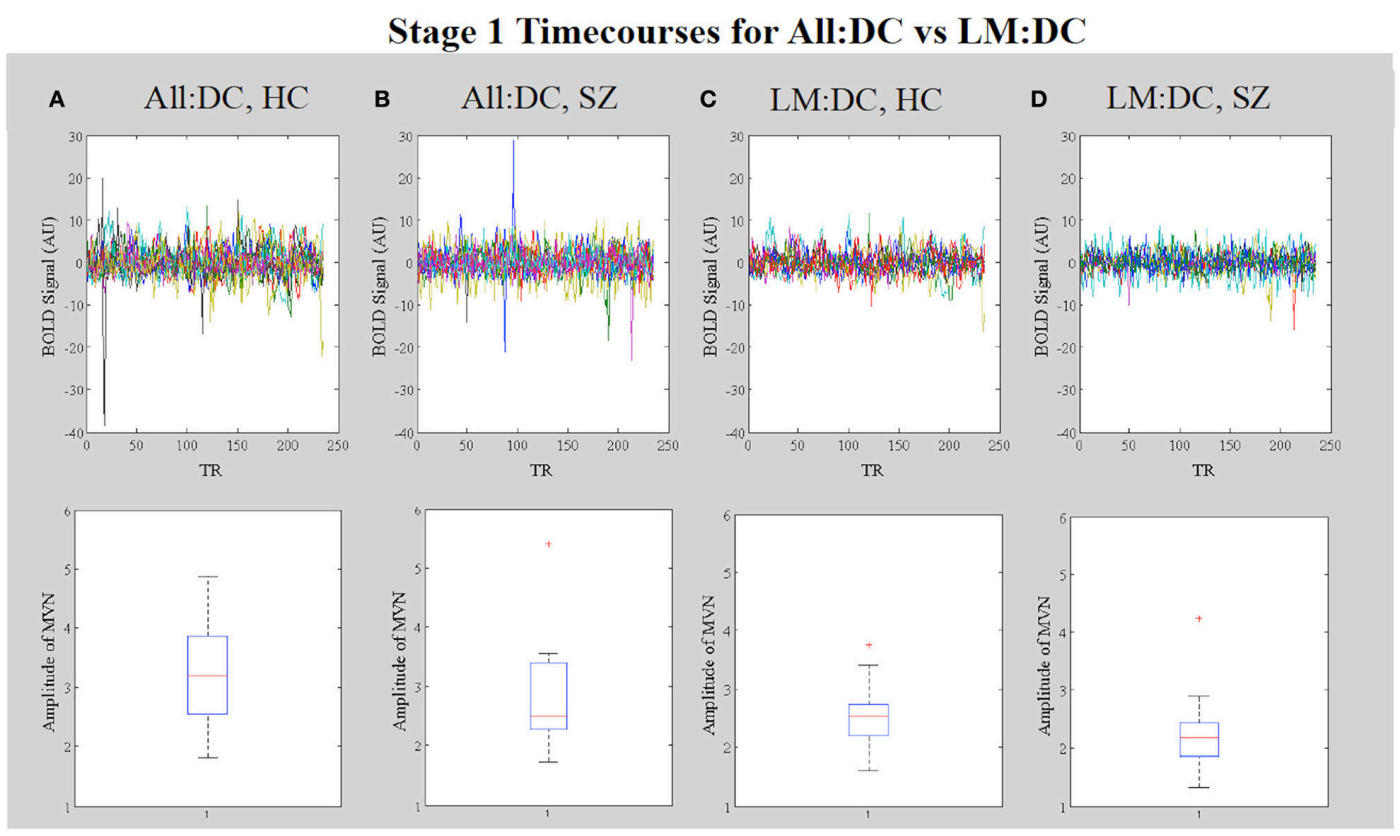
*****In Vivo*** Results**. Results. Stage 1 timecourses from dual regression can be used to identify corruption by motion. The upper four panels show the stage 1 timecourses for **(A)** All:DC HC, and **(B)** All:DC SZ subjects (which included subjects with larger motion effects in both groups). Timecourses for **(C)** LM:DC HC and **(D)** LM:DC SZ subjects, in which subjects with >1.5 mm motion were removed. The lower four panels show corresponding boxplots of the amplitudes of the timecourses for each group.

Inspection of the stage 1 timecourses for the MVN (Figure 13) shows that, in All:DC, the amplitudes of MVN timecourses are generally higher in HC vs. SZ (Figures 13A,B). After removing high motion subjects from the analysis, the within-group variability in the amplitudes is reduced (Figures 13C,D).

## Discussion

The GICA-DR approach is a powerful tool for identifying group differences in brain network functional connectivity that reflects differences in both network shape and network amplitude. Findings from our simulations based on creating ground truth group differences in network shape and amplitude from real resting state FMRI data show that:

1. Dual regression identifies subject-specific network timecourses with good temporal accuracy in the presence of amplitude and shape effects, assuming no subject variability in location of networks or network nodes.
2. ${\hat{B}}_{SM}$ SSSMs (e.g., raw regression coefficient maps from stage 2 of the dual regression without normalization of stage 1 timecourses) *should not* be used for inference at the group level because they are insensitive to network-wide amplitude effects, yet will mis-localize any within-network amplitude effects.
3. Using ${\hat{B}}_{SM}^{*}$ subject-specific spatial maps (SSSMs) from stage 2 of the dual regression (e.g., with normalization of stage 1 timecourses) for inference at the group level accurately localizes network-wide and within-network amplitude effects, as well as network shape differences.

While it may be tempting to use both ${\hat{B}}_{SM}$ and ${\hat{B}}_{SM}^{*}$ together to disambiguate amplitude effects (e.g., with the first being sensitive only to shape and the second sensitive to shape and network-wide amplitude effects), it is not recommended to do so because of the possibility of within-network amplitude effects. In this case, the ${\hat{B}}_{SM}$ maps will simply be incorrect. Since one cannot rule out the possibility of within-network amplitude effects without applying additional analyses, the implication is that within the GICA-DR framework, only *combined* amplitude and shape can be assessed. ${\hat{B}}_{SM}$ cannot be used to disambiguate the two effects. Thus, the important contribution of this paper is to show that design normalization of stage 1 timecourses is not a convenient method for teasing apart shape or network amplitude differences, it is a *necessary* step in the dual regression procedure to deal with any existing amplitude effects in a disciplined way to give group differences that are valid and localized correctly. That is, ${\hat{B}}_{SM}$ can't be used to infer shape differences since there is the potential to obtain mis-localized amplitude effects masquerading as connectivity differences in these maps. Another consequence of timecourse normalization is that amplitude effects cannot be studied separately from shape effects and vice-versa within the dual regression framework. On the other hand, resting state network amplitude has been shown to be an important network property that may be more interpretable relative to task FMRI, for which amplitude conveys meaningful information. Thus, including amplitude information together with shape can be a strength of the dual regression with timecourse normalization.

Our findings from the *in vivo* analyses illustrate that motion can mimic amplitude effects and that such affects can dramatically impact the connectivity maps, which is well-documented for seed-based connectivity analyses but less well-characterized for GICA-DR. Fortunately, the stage 1 timecourses are useful for identifying subjects with motion effects that have propagated into the connectivity analysis that could drive group differences, even motion effects that are not necessarily large and remain undetected during standard quality assurance. Using ${\hat{B}}_{SM}^{*}$ for inference on functional connectivity will not only accurately localize *real* network amplitude effects, because ${\hat{B}}_{SM}^{*}$ are also sensitive to motion-related amplitude effects, any motion effects will at least be accurately localized and thus not lead to spurious mis-localized group differences in other regions or networks that may be attributed to group differences in connectivity. That is, it is easier to detect motion-related amplitude effects in the group difference maps when these effects are accurately localized (with ${\hat{B}}_{SM}^{*}$), e.g., as rings around the brain edges or large differences at tissue interfaces, vs. being mis-localized throughout the image (with ${\hat{B}}_{SM}$), not giving the same visual appearance that is suggestive of motion artifacts.

In the context of other published findings, there have been a few studies that have assessed dual regression for back-reconstructing SSTC and SSSM for test-retest reliability and to assess performance in the presence of shape and amplitude differences (Zuo et al., 2010; Allen et al., 2012; Wang et al., 2013; Wisner et al., 2013). In the study by Zuo et al. (2010), they found that GICA-DR produced subject-specific brain network spatial patterns that had moderate-to-high reliability and reproducibility. They also reported that GICA-DR showed superior performance over applying single-subject ICA combined with template-matching procedures to identify the subject-specific brain networks. Wang et al. (2013) investigated the temporal signatures of RSNs that were generated from stage 1 of the dual regression and found that some univariate metrics such as entropy and the amplitude of low frequencies fluctuations (ALFF) of the stage 1 timecourses were reliable and reproducible. The neurometric profiles of RSNs identified using GICA-DR have also been shown to be highly reproducible and replicable across samples, and highly reliable within individuals for some RSNs (Wisner et al., 2013). For example, the right-lateralized fronto-parietal, auditory, and visuospatial networks had such a profile, suggesting that these networks are consistent with the profile of a trait. Other networks had a neurometric profile of high reproducibility and replicability, but low reliability in individual subjects. Left-lateralized fronto-parietal, emotion, and reward-related networks show this profile and, consequently, were interpreted as networks whose connectivity could change depending on the specific state of the individual.

Allen et al. (2012) investigated the ability of ICA combined with back-projection to investigate resting state networks in the presence of inter-subject variability in simulated FMRI data with spatial, amplitude, and temporal variability. They report that temporal concatenate GICA implemented with the GIFT toolbox combined with back-projection (Calhoun et al., 2001; http://icatb.sourceforge.net) and GICA-DR in FSL should generally perform extremely well for studying large-scale networks, but will be less effective when activations are very small in spatial extent in the presence of spatial variability. However, it is important to note that the general equivalence between the two approaches holds only for the case in which subject-level PCAs implemented in GIFT are not used for data reduction, only for whitening. If this is not the case, there is a potential for PCA bias (Beckmann, 2011).

While the GICA with back-projection in GIFT or dual regression in FSL may perform similarly under certain conditions in empirical studies, there is a very important distinction between back-projection techniques and dual regression. Back-projection techniques are heuristic approaches, [whose outcomes can depend strongly on the within-subject dimensionality reduction (Smith et al., 2014)] while the dual regression is a generative model that provides a statistical framework for assessing group differences in functional connectivity (Beckmann, 2011). As such, while back-projection approaches may be tuneable to give good results in empirical studies using specific conditions in simulated data (Erhardt et al., 2011; Allen et al., 2012), we advocate the dual regression approach as being theoretically well-principled and practically “safer.” The issue is not one of empirical validation, but one of assessing how the choice of parameters for the dual regression impacts the interpretability of the results with respect to group differences in functional connectivity.

One limitation of the current study is that, in the simulations, we investigated only one level of difference between groups in amplitude effects and shape; namely, we selected a network-wide amplitude difference of 10%, a within-network difference of 50% on one region of the network, and a difference in shape that reflected an absent connection of a region in one network in one group, with the region being connected to a different network in the other group. As such, our results do not speak to the sensitivity and specificity of the statistics we tested over a range of effects and signal levels, nor do our simulations incorporate spatial variability at the subject level. Our primary purpose in this work was to show that design normalization is necessary for dual regression to retain and reflect amplitude (either real network or motion-related) information accurately. There is evidence that spatial variability across subjects degrades the performance of DR for identifying subject-specific timecourses and spatial maps (Allen et al., 2012; Kim et al., 2012). While techniques such as iterated DR (Kim et al., 2012) may be necessary to derive accurate subject-specific timecourses and network maps, especially in the case of high-dimensional GICA for finer functional parcellations, the issue of amplitude effects at the DR stage must still be addressed. Simulating realistic resting state FMRI data with various ground truth sources of inter-subject variability can be used to further investigate these issues.

In sum, ${\hat{B}}_{SM}^{*}$ (from dual regression with normalization of stage 1 timecourses) accurately reflect both network amplitude and motion-related amplitude effects in group functional connectivity analyses. However, ${\hat{B}}_{SM}^{*}$ may require slightly larger sample sizes given that when these spatial maps are forward to the group analysis, they will have additional random effects variance from the amplitude that will make it more difficult to detect group differences. Inference using raw multiple regression coefficients (without normalization of stage 1 timecourses) can lead to mis-localized group differences, spurious group differences, or loss of sensitivity to real group differences. A stricter motion criterion is recommended for RSFC analyses, and inspection of the subject-specific timecourses from stage 1 of the dual regression is also recommended as part of the quality assurance to assess the effects of small movements (less than a single voxel) that may have large effects on the group difference maps. While we simply removed “high” motion subjects from the analysis, denoising the single-subject fMRI data using ICA (Salimi-Khorshidi et al., 2014) or using other approaches such as data scrubbing and regression of nuisance timecourses out of the data (Power et al., 2012; Siegel et al., 2014) prior to the dual regression or during dual regression (e.g., by including a subject's motion timecourses with stage 1 timecourses) may be desirable to remove motion effects and thus retain subjects. While these approaches have been shown to effectively remove motion, ICA-based denoising profoundly improves the reproducibility of group-level RSN spatial maps relative to either scrubbing or nuisance regression (Pruim et al., 2015). This is because the latter two approaches may also affect signals of interest, resulting in no improvement in reproducibility of group-level RSN spatial maps over doing no additional motion denoising, even though both techniques do effectively remove motion effects. Moreover, the fact that motion effects can mimic amplitude effects may be the fundamental reason that functional connectivity analyses are so sensitive to motion effects.

## Author contributions

LN, SS, and CB conceived and designed the study. LN conducted the simulations and *in vivo* data analysis, worked with co-authors to execute the study and interpret the findings, and wrote the paper. SS and DÖ contributed fMRI data for the simulations and the *in vivo* analysis, respectively. All authors contributed extensively to the interpretation of the findings and revising the manuscript.

## Funding

LN received support for this work from NIDA K25 DA017712 and NIDA R01 DA037265. DO received support from NIHM K24 MH104449.

### Conflict of interest statement

The authors declare that the research was conducted in the absence of any commercial or financial relationships that could be construed as a potential conflict of interest.

## Footnotes

^1^These output timecourses are *not* the same as the timecourses that are output from a GICA of resting state data using FSL MELODIC. The GICA timecourses from MELODIC are derived by inverting the initial PCA reduction done prior to the group ICA.

^2^All predictors are mean centered such that the mean of each predictor is zero, but the units are still on the original scale unless otherwise transformed.

^3^Our specific choices of RSNs, amplitude, and shape differences were based simply on demonstrating the ability of different statistics described in Section Introduction to accurately reflect amplitude and shape differences.
