# Supplementary material for: Using Dual Regression to Investigate Network Shape and Amplitude in Functional Connectivity Analyses
Source: Front Neurosci. 2017 Mar 13;11:115. doi: 10.3389/fnins.2017.00115 (PMC5346569; doi:10.3389/fnins.2017.00115)
Supplement: Supplementary file 1 [file DataSheet1.docx]

**Appendix I: Dual Regression With and Without Design Normalization**

Consider a network comprised of two voxels as shown in Fig. 14.A, with voxel V_i_, *i* = 1, 2. Also, assume the *T*-timepoint BOLD signal from each voxel in the network is generated from a single timecourse W shown in Fig. 14.B such that the timecourses are perfectly correlated, but with potentially different amplitudes, a_i_, at each voxel. The timeseries matrix Y (e.g., 2 voxels by T timepoints data matrix), which represents the two-voxel network in a single subject is generated as:

| $Y=AW+ E$ | Eq. 1 |
| --- | --- |
| $A= \left[ \begin{matrix} a_{1} \\ a_{2} \end{matrix} \right]$ | Eq. 2 |

W $\in\mathcal{R}^{1xT}$ with $\sigma_{W}=1$, and $E \sim N\left( 0,\sigma^{2}I \right)$ for simplicity. In this two-voxel network, $A$ is the true underlying spatial map with the amplitude of each voxel encoded in its values and voxel timecourses constructed for two cases: with $a_{1}=a_{2}=a$ to give a uniform network-wide amplitude equal to a constant (Fig. 14.C, a=2), and $a_{1}\neq a_{2}$ (Fig. 14.D, $a_{1}=1; a_{2}=2$) to give a within-network amplitude effect.

**Dual Regression**

Now consider a template network that will be used with the dual regression procedure to derive the spatial map and average timecourse of the network in our single subject’s FMRI data. Let the template spatial map be equal to $S=[1 1]'$, indicating that both voxels are included with equal weight in our template network. The goal of the dual regression is to identify the subject-specific network timecourse, which is a weighted sum of the timecourses of voxels in the subject-specific network, as well as the subject-specific spatial map corresponding to the template network from the subject’s FMRI data, Y.

Stage 1 of the dual regression is used to identify the subject-specific timecourse of the network, B_TC_:

| $Y=S{*B}_{TC}+E_{1}$ | Eq. 3 |
| --- | --- |

Solving for B_TC_:

| $\hat{B}_{TC}=pinv\left( S \right)*Y= \frac{a_{1}+ a_{2}}{2}*W$ | Eq. 4 |
| --- | --- |

Stage 2 of the dual regression is then used to obtain the subject-specific spatial map, $\hat{B}_{SM}$, as:

|  |  |
| --- | --- |
| $\hat{B}_{SM}=Y*pinv\left( \hat{B}_{TC} \right)$  which gives | Eq. 5 |
| $\hat{B}_{SM}=\left[ \begin{matrix} \frac{2a_{1}}{a_{1}+a_{2}} \\ \frac{2a_{2}}{a_{1}+a_{2}} \end{matrix} \right]$ | Eq. 6 |

Now consider two cases, a network with uniform network-wide amplitude effect, and a network with a within-network amplitude difference.

*Case 1: Uniform network-wide amplitude,* $a_{1}=a_{2}=a$

Application of dual regression in Eqs. 3-6 with $a_{1}= a_{2}$ and the “shape” of the timecourses all given by W will always give the same result, no matter the true amplitude of the network:

| $\hat{B}_{SM}=\left[ \begin{matrix} 1 \\ 1 \end{matrix} \right]$  In other words, a network-wide amplitude effect will not be represented in the estimated network spatial map, $\hat{B}_{SM}$ (which should be equal to A if amplitude is reflected in the stage 2 spatial maps) using standard dual regression. The implication in this case is that it is not possible to investigate group differences in uniform network amplitude with this approach. |
| --- |

*Case 2: Within-network amplitude difference,* $a_{1}\neq a_{2}$

If $a_{1}\neq a_{2}$, then there is a “within-network” amplitude difference in that one voxel timecourse in the network has greater amplitude than the timecourse from the other voxel. In this case, Eq. 5 gives the *wrong answer* for the estimated spatial map, $\hat{B}_{SM}\neq A$ (from Eq. 6), as each value in the estimated spatial map will contain amplitude information from *both* voxel timecourses and will thus not reflect any voxel’s true amplitude.

The implication in this case is that within-network amplitude effects are not accurately captured in the spatial maps output from stage 2 of the standard dual regression procedure. Furthermore, if any within-network amplitude effects are present, the estimated maps will be incorrect.

**Dual Regression with Design Normalization**

With design normalization, or normalizing $\hat{B}_{TC}$ to unit standard deviation, Eq. 5 becomes:

| $\hat{B}_{SM}^{*}=Y*pinv\left( \hat{B}_{TC}*D^{-1} \right)$ | Eq. 7 |
| --- | --- |

Where the star denotes with design normalization. D^-1^ is a matrix with the standard deviations (or amplitude) of $\hat{B}_{TC}$ from Eq. 4 on the diagonal, or in this case, simply $\sigma_{\hat{B}_{TC}}= \frac{a_{1}+a_{2}}{2}$ .

Which gives:

| $\hat{B}_{SM}^{*}=\left[ \begin{matrix} a_{1} \\ a_{2} \end{matrix} \right]$ | Eq. 8 |
| --- | --- |

The estimated spatial map in Eq. 8 contains the network-wide amplitude information, and is equal to A. In contrast to the solution in Eq. 5, the amplitude effects are correctly localized for each voxel. Thus, dual regression should *always* be implemented with design normalization. This will ensure that amplitude effects are accurately localized in the output spatial maps.


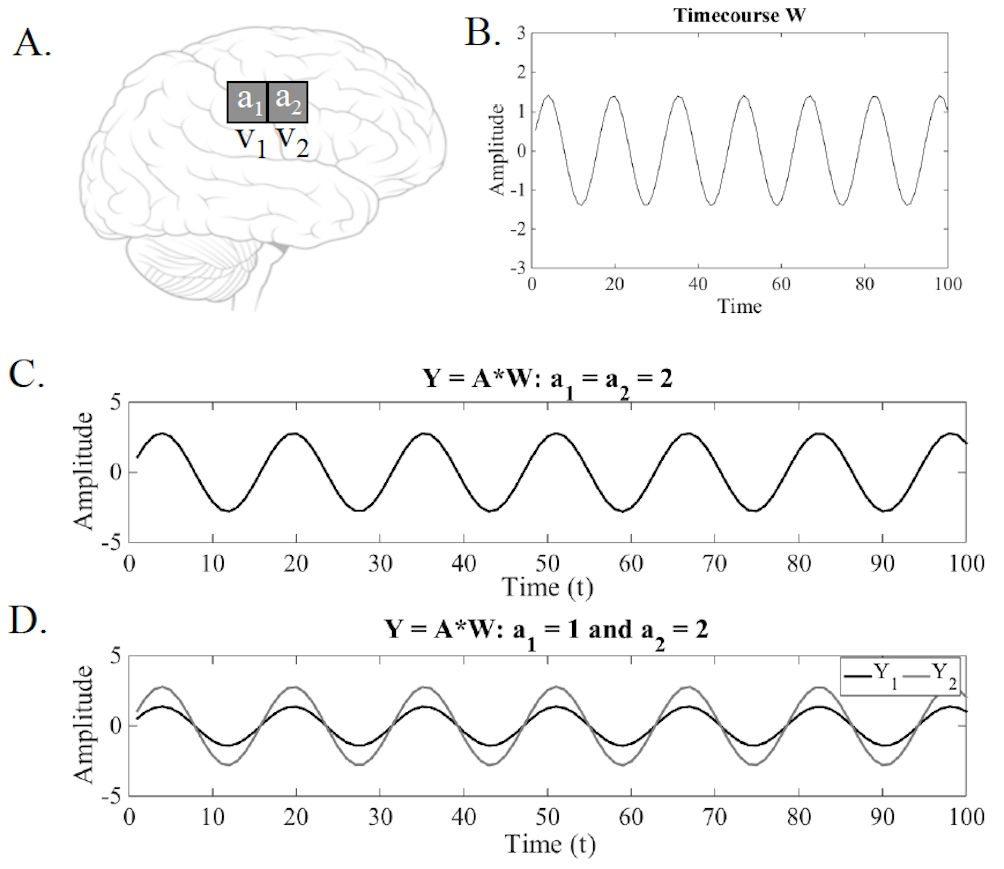


Supplementary Figure A) Network comprised of two voxels, V_i_, *i* = 1, 2. B) A T-timepoint BOLD signal from each voxel is generated from a single timecourse W. C) Uniform network-wide amplitude equal to a constant, a=2. D) Within-network amplitude effect with $a_{1}=1; a_{2}=2$.
